# Lineage dynamics of murine pancreatic development at single-cell resolution

**DOI:** 10.1038/s41467-018-06176-3

**Published:** 2018-09-25

**Authors:** Lauren E. Byrnes, Daniel M. Wong, Meena Subramaniam, Nathaniel P. Meyer, Caroline L. Gilchrist, Sarah M. Knox, Aaron D. Tward, Chun J. Ye, Julie B. Sneddon

**Affiliations:** 1 Diabetes Center, University of California, San Francisco, 513 Parnassus Avenue, San Francisco, CA 94143 USA; 2 Institute for Human Genetics, University of California, San Francisco, 513 Parnassus Avenue, San Francisco, CA 94143 USA; 3 Department of Cell and Tissue Biology, University of California, San Francisco, 513 Parnassus Avenue, CA 94143 USA; 4 Department of Otolaryngology-Head and Neck Surgery, University of California, San Francisco, 513 Parnassus Avenue, CA 94143 USA

## Abstract

Organogenesis requires the complex interactions of multiple cell lineages that coordinate their expansion, differentiation, and maturation over time. Here, we profile the cell types within the epithelial and mesenchymal compartments of the murine pancreas across developmental time using a combination of single-cell RNA sequencing, immunofluorescence, in situ hybridization, and genetic lineage tracing. We identify previously underappreciated cellular heterogeneity of the developing mesenchyme and reconstruct potential lineage relationships among the pancreatic mesothelium and mesenchymal cell types. Within the epithelium, we find a previously undescribed endocrine progenitor population, as well as an analogous population in both human fetal tissue and human embryonic stem cells differentiating toward a pancreatic beta cell fate. Further, we identify candidate transcriptional regulators along the differentiation trajectory of this population toward the alpha or beta cell lineages. This work establishes a roadmap of pancreatic development and demonstrates the broad utility of this approach for understanding lineage dynamics in developing organs.

## Abstract (web summary)

Coordinated proliferation and differentiation of diverse cell populations drive pancreatic epithelial and mesenchymal development. Here, the authors profile cell type dynamics in the developing mouse pancreas using single-cell RNA sequencing, identifying mesenchymal subtypes and undescribed endocrine progenitors.

## Introduction

**Fig. 1:**
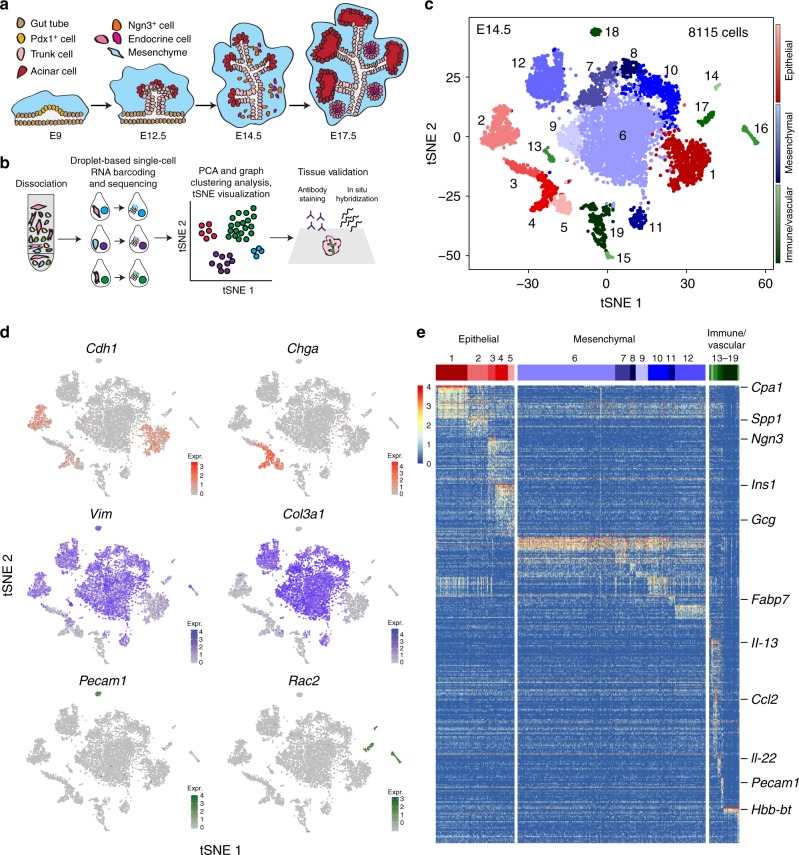
Single-cell sequencing identifies broad patterns of cellular heterogeneity in E14.5 murine pancreas. **a** Overview of murine pancreatic development. **b** Schematic of experimental approach. **c** t-Distributed stochastic neighbor embedding (t-SNE) visualization of populations from pooled E14.5 mouse pancreata (*n* = 14). Each dot represents the transcriptome of a single cell, color-coded according to its cellular identity (epithelial, mesenchymal, or immune/vascular). Each cell compartment contains multiple subpopulations, represented by varying degrees of color shading. **d** Established marker genes identify epithelial cells (Cdh1+), endocrine cells (Chga+), mesenchymal cells (Vim+ and Col3a1+), endothelial cells (Pecam1+), and immune cells (Rac2+). **e** Heatmap depicting greater than two-fold differentially expressed genes in each cluster compared to all other clusters. Cells are represented in columns, and genes in rows. Specific genes used to annotate clusters are indicated to the right of the heatmap

Pancreatic organogenesis is a complex and dynamic process that ultimately results in the generation of multiple cell lineages that perform the functions of the mature organ: the regulation of glucose homeostasis by the endocrine compartment and the production of digestive enzymes by the exocrine compartment. In the mouse, all known epithelial lineages of the pancreas derive from a small field of epithelial precursor cells within the foregut endoderm specified by the expression of *Pancreatic duodenal transcription factor 1* (*Pdx1*) (Fig. 1a)^1^. These Pdx1+ cells evaginate into a cap of surrounding mesenchymal cells around embryonic day 9 (E9), proliferate, and begin the process of branching morphogenesis. Further epithelial lineage diversification continues with the specification of Pdx1+ cells into tip and trunk domains by E12 and progresses to the restriction of tip cells to a digestive enzyme-producing acinar fate and of trunk cells to either a ductal or endocrine cell fate^1^. Within the trunk domain, induction of *Neurogenin 3* (*Ngn3*) expression defines the cells that will differentiate into one of five endocrine lineages: alpha, beta, delta, gamma, or epsilon cells, marked by expression of the hormones Glucagon (Gcg), Insulin (Ins), Somatostatin (Sst), Pancreatic polypeptide (PP), or Ghrelin (Ghrl), respectively^2^. Gastrin+ cells have also been recently described^3^. Despite previous work focused on the formation of the endocrine compartment, the precise timing and coordination of lineage decisions are not completely understood.

Although the pancreatic mesenchyme is required for the proper differentiation, proliferation, and morphogenesis of the epithelial network^1^, little is known about the cell identities and lineages that compose the pancreatic mesenchyme during development. Even less is known about the mechanisms by which these distinct mesenchymal cell types interact with one another and with the cells of the epithelial compartment during development and in the adult organ. Therefore, a deeper understanding of the full diversity of the mesenchymal cell types, as well as their global gene-expression profiles, will serve as a basis for understanding these key cellular interactions.

Recent studies of late embryonic, postnatal, and adult alpha and beta cells have demonstrated the power of single-cell transcriptomic profiling for unraveling endocrine lineage heterogeneity and revealing distinct transcriptional states of beta cell maturation^4–6^. Here, we perform droplet-based, single-cell RNA sequencing of entire murine embryonic pancreata at earlier developmental timepoints to describe the cellular diversity and dynamics of gene expression in both the epithelial and mesenchymal compartments. We further identify and validate these populations within mouse and human pancreatic tissue, as well as human embryonic stem cell (hESC)-derived endocrine progenitor cells. Finally, we predict lineage relationships, identify previously unappreciated intermediate progenitor cells, and validate our methodology using in vivo genetic lineage tracing.

## Results

### Cellular heterogeneity in the murine pancreas

We first characterized the major sources of cellular heterogeneity in the developing pancreas. Two batches of mouse pancreata at E14.5, a particularly active time of expansion, morphogenesis, and diversification^2^ (Fig. 1a), were dissected from individual litters, dissociated into single-cell suspensions, sorted for live cells, and sequenced using the 10x Chromium Single-Cell version 1 (v1) kits (Fig. 1b and Supplementary Fig. 1a–e). We performed filtering, normalization, variable gene identification, linear regression for batch, and principal component analysis (PCA) with the R package Seurat (Supplementary Fig. 1d, e and 2a, b). Graph-based clustering^7^ of batch-adjusted, merged data identified 19 distinct cell populations, classified as epithelial, mesenchymal, immune, or vascular populations based on the expression of known markers (Fig. 1c, d and Supplementary Data 1). We identified expected populations, including endocrine, exocrine (acinar and ductal), and endothelial cells (Fig. 1e). The proportions of endocrine, mesenchymal, immune, and vascular populations were similar between E14.5 batches (Supplementary Fig. 2b–d). Downsampling analysis confirmed that sufficient sequencing depth had been reached for calling clusters (Supplementary Fig. 2e–g). These results reveal the power of single-cell RNA sequencing to identify a broad range of cell types during development.

### Characterization of mesenchymal heterogeneity

**Fig. 2:**
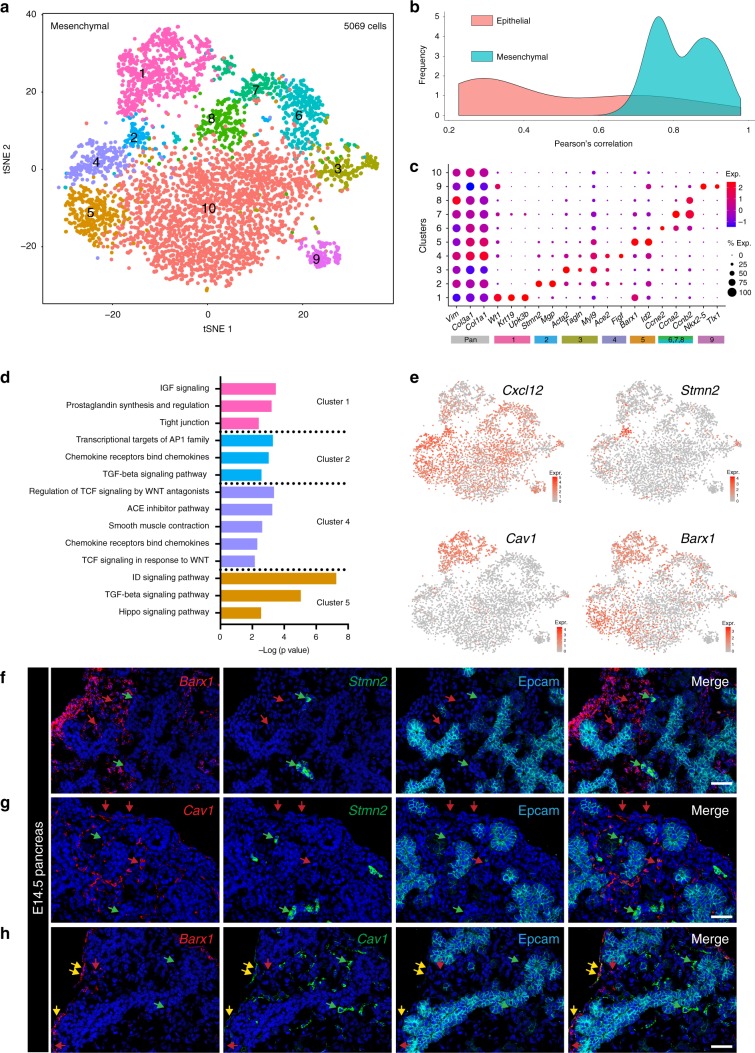
Identification of multiple uncharacterized mesenchymal populations. **a** t-SNE visualization of subclustered E14.5 mesenchymal clusters (from *n* = 14 pancreata). **b** Density plot depicting Pearson’s correlation values (depicted in heatmap in Supplementary Fig. 3b) within the epithelial and mesenchymal populations based on average gene expression in each cluster. **c** Dot plot of top differentially expressed markers of each mesenchymal population. Bars are color-coded by cluster identity in **a**. The gray bar represents pan-mesenchymal markers. The size of each dot represents the proportion of cells within a given population that expresses the gene; the intensity of color indicates the average level of expression. **d** Pathway analysis of genes greater than 2-fold differentially expressed by cells in clusters 1, 2, 4, and 5. **e** Expression of genes marking clusters 1 (*Cav1*), 2 (*Stmn2*), 4 (*Cxcl12*), and 5 (*Barx1*) in all E14.5 mesenchymal cells. Color intensity indicates level of expression. **f**–**h** Multiplexed fluorescent ISH combined with Epcam IF validates clusters 2 and 5 (**f**) and cluster 1 (**g-h**). Epcam marks pancreatic epithelium. In (**f**), Barx1 + cells (red arrows, cluster 5) are distinct from Stmn2+ cells (green arrows, cluster 2), validating the single-cell data. In (**g**), Cav1+ cells (red arrows, cluster 1) are distinct from Stmn2+ cells (green arrows, cluster 2). In (**h**), Barx1+ cells that do not express *Cav1* (red arrows) represent cluster 5, whereas Barx1+/Cav1+ cells (yellow arrows) represent cluster 1. Cav1+ cells that do not express *Barx1* are also identified (green arrows), likely representing endothelial cells^79^. Scale bar represents 50 µm in **f–h**

While previous studies have identified numerous markers of pancreatic epithelial populations^2^, comparatively little is known about heterogeneity among pancreatic mesenchymal cells. We characterized the mesenchymal compartment by subclustering only mesenchymal cells (5069 cells) and reperforming the clustering analysis (Fig. 2a and Supplementary Fig. 3a). Despite being less divergent from one another than were cells in the epithelial compartment (Fig. 2b and Supplementary Fig. 3b), mesenchymal cells could still be subdivided into ten transcriptionally distinct mesenchymal clusters (Fig. 2a, c and Supplementary Data 2). We verified the differential gene-expression analysis with three tests: bimodal likelihood ratio test^8^, Wilcoxon rank sum, and MAST^9^ (Supplementary Fig. 3c and Supplementary Data 2). We annotated two clusters based on the expression of known marker genes: cluster 1 is pancreatic mesothelial cells (*Wt1*, *Krt19*, and *Upk3b*)^10,11^ and cluster 3 represents vascular smooth muscle (VSM) cells (*Acta2*, *Tagln*, and *Myl9*) (Fig. 2c and Supplementary Data 2)^12^. Indeed, in E14.5 pancreas, Wt1 expression was restricted to the tissue edge, as expected for mesothelial cells, while Acta2 expression was localized to cells surrounding vessels, as expected for VSM cells (Supplementary Fig. 3d, e). Cells in the mesothelial cluster also expressed the secreted factors *Fgf9*, *Pdgfc*, *Rspo1, and Igfbp5* (Supplementary Fig. 3f) and genes regulating prostaglandin hormone signaling and tight junctions (Fig. 2d and Supplementary Data 3).

The remaining mesenchymal clusters included proliferating cells (clusters 6–8), a large cluster (10) expressing pan-mesenchymal markers, and four clusters (2, 4, 5, and 9) each expressing a signature distinct from that of cluster 10 (Fig. 2a, c and Supplementary Data 2). Cluster 2 was defined by differential expression of *Stathmin 2* (*Stmn2*), a gene involved in neurite outgrowth and osteogenesis^13,14^. We also found two populations, clusters 4 and 5, that differentially expressed multiple secreted factors. Cluster 4 expressed *Ace2*, the chemokines *Cxcl12* and *Cxcl13*, and *Vegfd*, while cluster 5 expressed high levels of the Wnt antagonists *Secreted frizzled-related protein 1 and 2* (*Sfrp1* and *Sfrp2*) (Fig. 2c–e and Supplementary Data 2). Cluster 5 also expressed the transcription factor *BarH-like homeobox1 1* (*Barx1*) and members of the Id DNA-binding protein family (Fig. 2c–e and Supplementary Data 2). Cluster 9 expressed *Nk2 homeobox 5* (*Nkx2-5*) and *Tlx1*, transcription factors reported to contribute to splenic development during a window in which the embryonic pancreas and spleen share a mesenchymal compartment (Fig. 2c)^15^. Pathway analysis identifies multiple signaling pathways that may be functionally relevant in these populations (Fig. 2d and Supplementary Data 3). We validated a subset of these distinct clusters using dual in situ hybridization/immunofluorescence (ISH/IF) on E14.5 pancreas for differentially expressed markers of clusters 1 (*Cav1* and *Barx1*), 2 (*Stmn2*), and 5 (*Barx1*) (Fig. 2e–h). These gene-expression profiles demonstrate a previously underappreciated level of heterogeneity in the mesenchymal compartment of the developing pancreas.

### Mesothelial cells undergo changes across developmental time

**Fig. 3:**
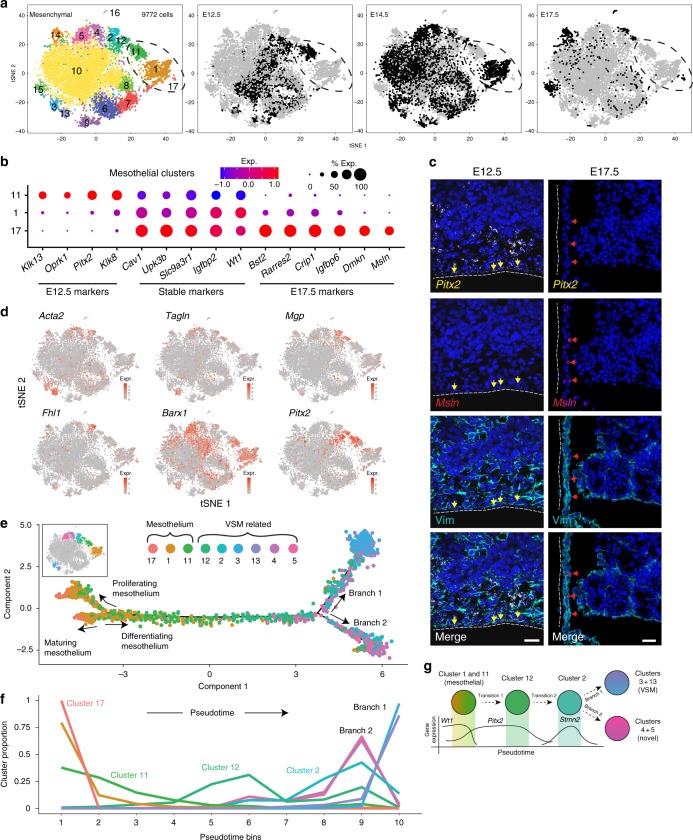
Mesothelial cells are dynamic over developmental time and are predicted to give rise to vascular smooth muscle populations. **a** t-SNE visualization of merged mesenchymal clusters from E12.5 (*n* = 18 pancreata), E14.5 (*n* = 14 pancreata for batch 1; *n* = 11 for batch 2), and E17.5 (*n* = 8 pancreata) tissue. Mesenchymal clusters were identified at each timepoint, subclustered, merged together, and reanalyzed. Cells are colored by cluster or timepoint. Dotted circle highlights timepoint-segregated mesothelial clusters. **b** Dot plot of top differentially expressed genes in timepoint-specific mesothelial clusters (clusters 1, 11, and 17). Size of the dot represents proportion of the population that expresses each specified marker. Color indicates level of expression. **c** ISH for *Pitx2* and *Msln* in E12.5 and E17.5 pancreata. *Pitx2* expression was detected in E12.5, but not E17.5 mesothelium, whereas *Msln* was detected in E17.5, but not E12.5 mesothelium. Vimentin (Vim) IF staining depicts pancreatic mesenchyme. Dotted line indicates tissue boundary. Yellow arrows identify Pitx2+ mesothelial cells. Red arrows identify Msln+ mesothelial cells. Scale bar represents 50 µm. **d** Expression levels of VSM-related genes in merged mesenchymal clusters. Color intensity indicates level of expression. **e** Pseudotime ordering of mesothelial and VSM-related merged mesenchymal clusters. Colors correspond to t-SNE in **a**. All clusters are individually plotted in Supplementary Fig. 3j. **f** Cluster proportions over pseudotime. Pseudotime was binned into ten groups and the proportion of each cluster within that bin of pseudotime was calculated. **g** Model of lineage relationships among mesothelial and VSM-related mesenchymal populations based on pseudotime ordering in **e**

During organogenesis, the dynamics of each lineage are defined by the expansion, differentiation, and maturation of its constituent cells. To address how these processes change across chronological time within the developing pancreas, we performed single-cell sequencing at two additional timepoints, E12.5 and E17.5 (Fig. 3a). We identified mesenchymal cells from E12.5, E14.5, and E17.5 timepoints, merged them into one dataset, and reperformed the clustering analysis. We identified the clusters detected in our E14.5 analysis (clusters 1–10) along with seven new clusters (11–17) (Fig. 3a, Supplementary Fig. 3g–i, and Supplementary Data 4). The addition of E12.5 and E17.5 cells revealed further sub-division of the mesothelium into timepoint-specific clusters (1, 11, and 17), each with unique transcriptomic signatures (Fig. 3a, b). Within the mesothelium, we verified *Paired-like homeodomain transcription factor 2* (*Pitx2*) expression at E12.5 and its absence at E17.5 and *Mesothelin* (*Msln*) expression at E17.5 and its absence at E12.5 (Fig. 3c), consistent with the single-cell data. These data provide evidence of transcriptional maturation over developmental time within the mesothelial compartment.

While the mesothelium is a well-established mesenchymal progenitor cell population for VSM and fibroblasts in multiple other organs, both the role of the mesothelium and the origin of the mesenchymal cell types within the pancreas remain uncharacterized^16–19^. We utilized our single-cell mesenchymal dataset to determine whether the pancreatic mesothelium may function as a mesenchymal progenitor cell population during development. We found six populations (clusters 2, 3, 4, 5, 12, and 13) that expressed VSM cell genes, such as *Acta2* and *Tagln*, or genes known to regulate VSM development, such as *Mgp*^20^, *Fhl1*^21,22^, *Barx1*^23^, and *Pitx2*^24^ (Fig. 3d). Based on these VSM-related gene-expression profiles, we hypothesized that these populations could represent VSM progenitors derived from the pancreatic mesothelium. To test the lineage relationships among these populations, we ordered cells in pseudotime based on their transcriptional similarity^25^. This analysis placed mesothelial cells on one side of the pseudotime trajectory (Fig. 3e). Mesothelial branches corresponded to either a maturation process, based on placement of E17.5 cells at the branch terminus, or proliferating mesothelium, based on expression of proliferation genes (Fig. 3e and Supplementary Fig. 3j). VSM-related populations were placed on the other side of the trajectory (Fig. 3e and Supplementary Fig. 3j). We calculated the proportion of each population over pseudotime and found a transition from the E12.5 mesothelial population (cluster 11) to cluster 12, both of which share expression of the gene *Pitx2* (Fig. 3e–g). Cluster 12 then transitioned into the *Stmn2*-expressing cluster 2, which split into a branch composed of VSM populations, clusters 3 and 13 (Branch 1), and a branch composed of clusters 4 and 5 (Branch 2) (Fig. 3e–g). Thus, this analysis proposes clusters 2 and 12 as potential mesothelial-derived mesenchymal progenitor populations that can contribute to the VSM lineages (Fig. 3g). Our analysis has identified and validated multiple mesenchymal subtypes and possible lineage relationships among them.

### A previously undescribed endocrine progenitor population

**Fig. 4:**
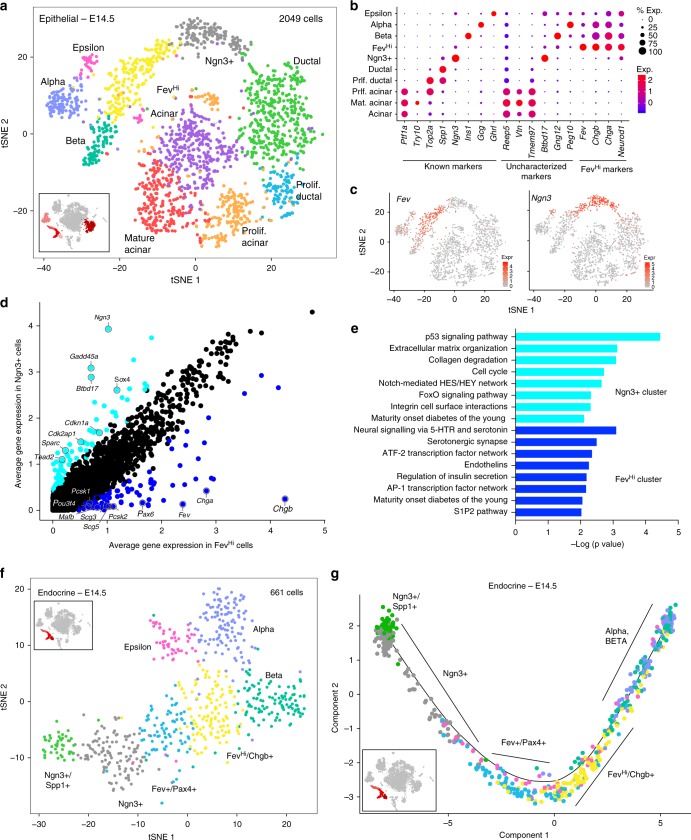
Identification of epithelial cell populations in E14.5 mouse pancreas. **a** t-SNE visualization of epithelial groups only, as defined in Fig. 1. **b** Dot plot depicting known and uncharacterized markers of epithelial populations, as well as markers specific to the Fev^Hi^ population. Size of the dot represents proportion of the population that expresses each specified marker. Color indicates level of expression. **c** Expression of *Fev* and *Ngn3* within epithelial cells. Color indicates level of expression. **d** Gene expression comparison between the Ngn3+ and Fev^Hi^ population. Genes greater than 2-fold differentially expressed are highlighted in dark blue (higher in Fev^Hi^ cells) or light blue (higher in Ngn3+ cells). **e** Pathway analysis of genes greater than 2-fold differentially expressed in Ngn3+ and Fev^Hi^ populations. **f** t-SNE visualization of the 661 cells of the endocrine lineage (Ngn3+, Fev^Hi^, alpha, beta, and epsilon populations). **g** Pseudotime ordering of Ngn3+, Fev+/Pax4+, Fev^Hi^, alpha, and beta cell populations place Fev+ cells between Ngn3+ and hormone+ populations

After assessing the heterogeneity within the mesenchymal compartment, we next focused on the epithelial cells. We first subclustered the 2049 cells from our E14.5 dataset that comprised just the epithelial populations (Fig. 4a and Supplementary Fig. 4a). We identified 10 clusters, including acinar, ductal, beta, alpha, and Ngn3 + progenitor populations, as revealed by differential expression of known markers (Fig. 4a, b, Supplementary Fig. 4b, and Supplementary Data 5). Our analysis highlighted previously uncharacterized markers of acinar, Ngn3+, beta, and alpha cell populations, such as *Reep5*, *Btbd17*, *Gng12*, and *Peg10*, respectively (Fig. 4b and Supplementary Data 5). We also found *Sst*- and *Pancreatic polypeptide* (*Ppy*)-expressing cells, but they did not cluster into their own populations (Supplementary Fig. 4c).

After the ductal, acinar, Ngn3+, and hormone+ populations had been accounted for, there still remained one population that eluded classification based on known marker genes. This population was distinguished from all other epithelial populations by high-level expression of the E26 transformation-specific transcription factor *Fev*, previously shown to be expressed within the developing pancreas but not described as a marker of a distinct epithelial population^26^ (Fig. 4a, b and Supplementary Data 5). This Fev+ population expressed genes marking endocrine lineage cells, such as *Paired box 4* (*Pax4*), *chromogranins A/B (Chga/b)* and *Neurod1*^1^ (Supplementary Fig. 4d), but not mature endocrine markers, such as *Insulin1* (*Ins1*) or *Gcg*, or the transitory early endocrine lineage marker, *Ngn3* (Fig. 4b, c and Supplementary Data 5). Pairwise comparison between the Fev+ and Ngn3+ clusters identified 99 genes more highly expressed in Fev+ and 87 more highly expressed in Ngn3+ cells, suggesting that they are distinct populations (Fig. 4d). This Fev+, Ngn3−, hormone− cluster will henceforth be referred to as the Fev^Hi^ population. Pathway analysis of the Ngn3+ and Fev^Hi^ populations revealed enrichment of cell cycle and Notch signaling pathways in Ngn3+ cells (Fig. 4e and Supplementary Data 6), likely reflecting the exit of Ngn3+ progenitors from the cell cycle^27^ and the role of *Ngn3* in Notch signaling^28^. The Fev^Hi^ cluster expressed genes in pathways related to serotonin, insulin, Activating transcription factor 2 (Atf2), and sphingosine-1-phosphate signaling, which have been reported to regulate endocrine differentiation^29,30^. This relationship to serotonin is consistent with prior work establishing *Fev* as a critical transcription factor in serotonergic neurons^26,31^.

Further subclustering of all 661 cells within the endocrine lineage revealed additional sub-groups of *Fev*-expressing cells. The first was marked by high expression of *Pax4* and *Runx1 Translocation Partner 1* (*Runx1t1*) and lower levels of *Ngn3*. The second was marked by *Chgb* and *Vimentin* (*Vim*) (Fig. 4f, Supplementary Fig. 4e, f, and Supplementary Data 7). Therefore, our analysis proposed the existence of multiple intermediate states, marked by *Fev*, within the endocrine lineage. The *Fev* gene was also expressed at lower levels in a subset of the hormone-producing alpha, beta, and epsilon cell populations, which will collectively be referred to as hormone+/Fev^Lo^ populations (Fig. 4b).

Given that the Fev+ populations expressed endocrine lineage genes, we utilized pseudotime ordering^25^ to test the hypothesis that both Fev+ populations were lineage-related to the Ngn3+ progenitors that give rise to the endocrine compartment of the pancreas^32^. This de novo reconstruction of the developmental trajectory placed both the Fev+/Pax4+ and Fev^Hi^/Chgb+ cells between Ngn3+ endocrine progenitors and alpha and beta cells (Fig. 4g and Supplementary Fig. 4g), suggesting that Fev^Hi^ cells comprise a progenitor stage following *Ngn3* expression and before hormone acquisition. The Fev+/Pax4+ population was placed closer in pseudotime to the Ngn3+ population and was followed by the Fev^Hi^/Chgb+ population (Fig. 4g), indicating that the former represents an earlier cell state. Unlike alpha and beta cells, epsilon cells were found throughout the trajectory populated by the Fev+/Pax4+ and Fev^Hi^/Chgb+ populations (Fig. 4g), possibly reflecting their function as multipotent progenitor cells for alpha and gamma lineages during development^33^.

**Fig. 5:**
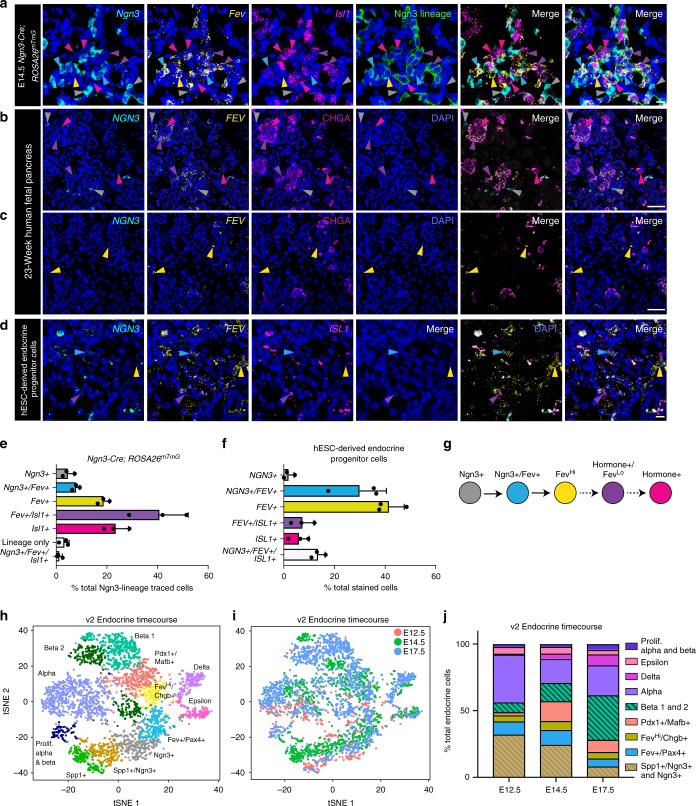
Fev^Hi^ cells are endocrine progenitors. **a** In situ hybridization (ISH) for *Ngn3*, *Fev*, and *Isl1* in lineage-traced *Ngn3-Cre; Rosa26*^*mTmG*^ E14.5 pancreata where *Ngn3*-lineage-traced cells are mGFP+. Gray arrowheads identify *Ngn3*+ cells, presumably not yet Ngn3-lineage labeled due to the transient nature of *Ngn3* expression and the delay of Cre-mediated recombination that permits expression of mGFP. Blue arrowheads identify *Ngn3*+/*Fev*+ cells that are Ngn3-lineage-traced. Yellow arrowheads identify *Ngn3*-lineage-traced cells that are *Fev*+, but do not express *Ngn3* or *Isl1*. Purple arrowheads identify *Fev*+/*Isl1*+ cells that are Ngn3-lineage-traced. Magenta arrowheads identify *Isl1*+ cells that are Ngn3-lineage-traced. **b**–**c** Dual ISH/immunofluorescence (IF) for *NGN3* and *FEV* mRNA and CHGA protein in human fetal pancreas at 23 weeks of gestation (*n* = 1 pancreas). Gray arrowheads identify *NGN3*+ cells. Yellow arrowheads identify *FEV*+ cells. Purple arrowheads identify *FEV*+/CHGA+ cells. Magenta arrowheads identify CHGA+ cells. **d** Multiplexed fluorescent ISH for *NGN3*, *FEV*, and *ISL1* mRNA in hESC-derived endocrine progenitor cells. Blue arrowheads identify *NGN3*+/*FEV*+ cells. Yellow arrowheads identify *FEV*+ cells. Purple arrowheads identify *FEV*+/*ISL1*+ cells. **e** Quantification of each population detected in *Ngn3*-lineage-traced pancreata as a percentage of *Ngn3*-lineage-traced cells (*n* = 464 cells, 6 pancreata). Data are represented as mean + standard deviation (SD). **f** Quantification of each population detected in hESC-derived progenitor cells as a percentage of total stained cells (*n* = 418 cells, 3 clusters representing technical replicates from one hESC differentiation). Data are represented as mean + SD. **g** Proposed model for the derivation of Fev^Hi^ endocrine cells from Ngn3+ cells, and their differentiation into hormone+/Fev^Lo^ endocrine cells. Colors of arrowheads and bars in **a**–**f** correspond to cell identity in **g**. **a**, **d** Scale bar: 10 µm. **b**, **c** Scale bar: 20 µm. **h** t-SNE visualization of v2 merged endocrine timecourse (E12.5, E14.5, aggregated E17.5). Clusters are annotated based on correlation with v1 dataset or top differentially expressed genes. **i** Timepoint labels for v2 merged endocrine timecourse data. t-SNE is the same as **h**. **j** Cell type proportions at each timepoint, calculated from the clusters depicted in **h**

To validate these lineage relationships, we performed an in vivo lineage trace of Ngn3+ cells. In E14.5 *Ngn3-Cre; ROSA26*^*mTmG*^ mouse pancreata, where lineage-traced cells are membrane-green fluorescent protein (GFP+)^34^, approximately 20% of all *Ngn3*-lineage-traced cells were identified as the Fev^Hi^ population by the presence of *Fev* and the absence of both *Ngn3* and the pan-differentiated endocrine cell marker *Islet1* (*Isl1*) (Fig. 5a, e, yellow arrows and bar, and Supplementary Fig. 4h). We also detected the hormone+/Fev^Lo^ population identified by our single-cell data (Fig. 5a, purple arrows) and cells that co-expressed *Fev* and *Ngn3* (blue arrows), consistent with a model in which Fev^Hi^ cells represent an intermediate progenitor state following Ngn3+ cells, but prior to differentiated endocrine cells (Fig. 5g).

We next tested if the Fev^Hi^ population was also present in developing human pancreatic tissue. In human fetal pancreas at 23 weeks postconception, we observed cells that expressed only *NGN3* (Fig. 5b, gray arrows), only CHGA (magenta arrows), a marker of all hormone-expressing endocrine cells, and both *FEV* and CHGA (purple arrows). We also detected cells that expressed *FEV* but not *NGN3* or CHGA (Fig. 5c, yellow arrows), analogous to the murine Fev^Hi^ population. The existence of these cellular states in human development suggests that the lineage relationships we identified generalize beyond murine pancreatic organogenesis to that of human, as well.

We then probed hESCs undergoing directed differentiation toward the pancreatic beta cell lineage in vitro^35^. *FEV* was detected in endocrine progenitor stage cells and beta-like cells (BLCs) at levels comparable to adult human islets, but not in undifferentiated hESCs (Supplementary Fig. 4i). Further, we observed *FEV*+ (*NGN3*−/*ISL1*−) (yellow arrows), *FEV*+/*ISL1*+ (*NGN3*−) (purple arrows), and *NGN3*+/*FEV*+ (*ISL1*−) (blue arrows) populations in differentiating hESC-derived cells midway through the endocrine progenitor stage (Fig. 5d, f). While endocrine differentiation progresses as a wave throughout development^36^ in vivo, it is more synchronized in the hESC differentiation platform in vitro^35,37,38^. At a timepoint directly preceding beta cell differentiation, we found that nearly 70% of hESC-derived cells were either *NGN3*+/*FEV*+ or *FEV*+ (Fig. 5f, blue and yellow bars). These data place the FEV+ population at a timepoint consistent with an endocrine progenitor population during human beta cell differentiation in vitro.

### Endocrine dynamics over developmental time

Although we had captured comparatively fewer epithelial cells at E12.5 and E17.5 than at E14.5, we could still identify the Fev^Hi^ cells at both timepoints (Supplementary Fig. 5a). To capture more epithelial cells and account for those that were missing from E12.5 and E17.5 version 1 (v1) runs, we reperformed an entirely new (version 2) set of single-cell RNA-sequencing experiments at E12.5, E14.5, and E17.5 after depletion of CD140a+ mesenchymal cells in order to enrich for epithelial cells (Supplementary Fig. 5b, c). Given the high numbers of red blood cells at E17.5, we ran two wells of E17.5 cells (replicates 1 and 2) to increase our capture of epithelial cells and then aggregated the datasets. We first analyzed the exocrine compartment and identified acinar, ductal, and proliferating populations of both at all timepoints (Supplementary Fig. 5d and Supplementary Data 8–10). We then focused on the endocrine compartment, where we captured 584, 1267, and 1837 endocrine cells at E12.5, E14.5, and E17.5, respectively. We found similar gene-expression topologies as in our v1 dataset but gained additional resolution with increased cell numbers and transcriptomic coverage (Supplementary Fig. 5e and Supplementary Data 11–13).

To analyze how endocrine populations change over time, we merged all three v2 timepoints into one dataset using canonical correlation analysis^39^. We correlated the v2 dataset to the v1 dataset and could identify all populations present in the v1 dataset (Supplementary Fig. 5f and Supplementary Data 14). We also found additional populations, including a cluster characterized by decreased expression of *Fev* and increased expression of *Pdx1* and *Mafb*, genes with known roles in endocrine lineage decisions (Fig. 5h and Supplementary Fig. 5g). This Pdx1+/Mafb+ population correlates most strongly with the Fev^Hi^/Chgb+ population, as well as both the alpha and beta cell populations in the v1 dataset (Supplementary Fig. 5f). We also found a second beta cell population characterized by increasing expression of *Ins1* and *Ins2* and lower expression of *Pdx1*, perhaps representing more mature beta cells (Supplementary Fig. 5g). Indeed, this second beta cell group is almost entirely comprised of cells from the E17.5 timepoint (Fig. 5i). To examine how these populations shift over developmental time, we calculated the proportion of these populations at each timepoint (Fig. 5j). We found shifts in cell proportions that match those reported in literature, such as a high proportion of alpha cells early in development at E12.5 and increasing proportions of beta and delta cells at later timepoints^36^. The Ngn3+ population decreased over time, while the Fev+/Pax4+, Fev^Hi^/Chgb+, and Pdx1+/Mafb+ populations peaked at E14.5, consistent with previous studies that reported peak *Ngn3* expression at approximately E14.5 and its subsequent downregulation as differentiation into endocrine lineage ensues^40^. At E17.5, we also found an increasing proportion of proliferating endocrine cells, presumably those responsible for the expansion of endocrine cell mass in later embryonic development^41^. These results from the larger v2 dataset confirm our initial findings from the v1 dataset and add additional resolution to the endocrine populations during pancreatic development.

### Lineage decisions within the endocrine compartment

**Fig. 6:**
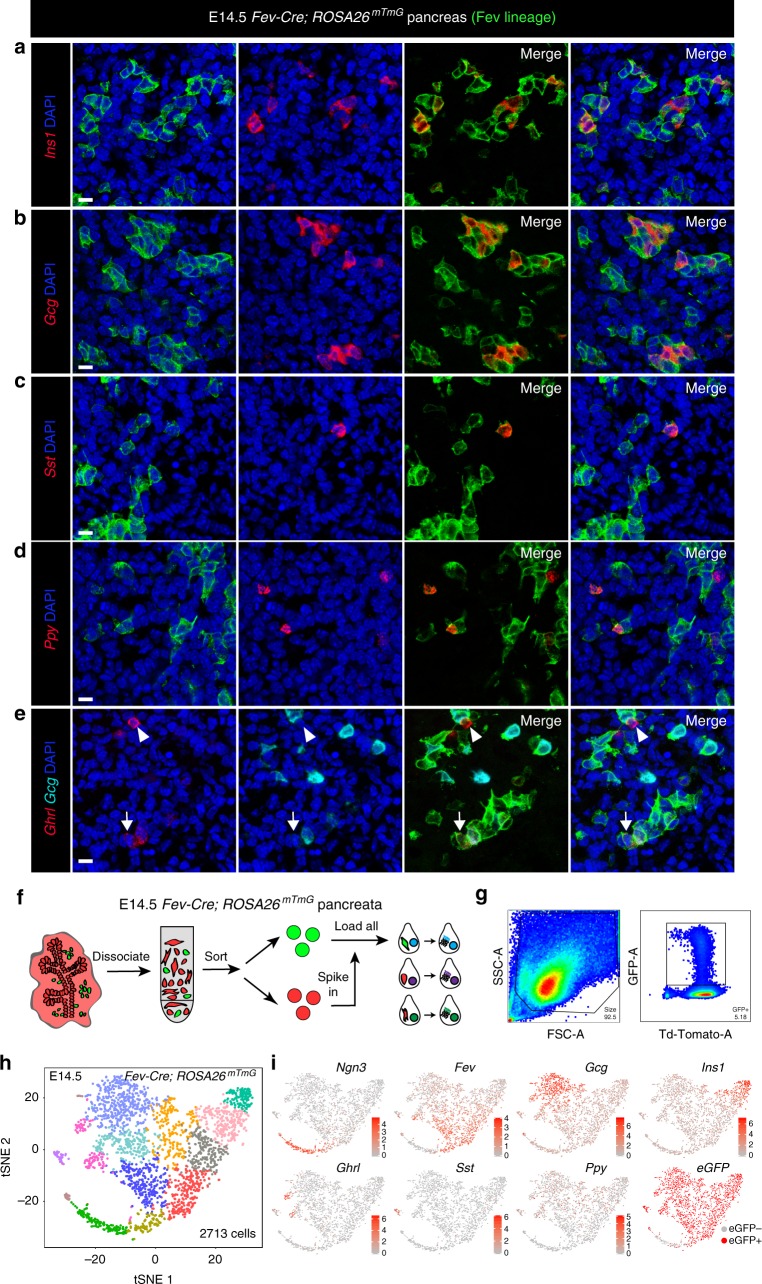
Differentiated hormone+ endocrine cells transit through a *Fev*-expressing stage during pancreatic development. **a**–**e** Dual IF (for membrane GFP) and fluorescent ISH for hormones in *Fev-Cre; ROSA26*^*mTmG*^ lineage-traced animals at E14.5. *n* = 46 cells of 4 pancreata for *Ins1* (100% labeled-lineage); *n* = 103 of 4 pancreata cells for *Gcg* (100% lineage-labeled); *n* = 6 cells of 2 pancreata for *Sst* (100% lineage-labeled); *n* = 26 cells of 2 pancreata for *Ghrl/Gcg* (23.2% lineage-labeled); *n* = 71 cells of 8 pancreata for *Ppy* (90.1% lineage labeled). Scale bar represents 10 µm. **f** Schematic of E14.5 *Fev-Cre; ROSA26*^*mTmG*^ FACS sorting and single-cell RNA sequencing. **g** Representative FACS plots of sorted single, live GFP+ and TdTomato+/GFP− cells from dissociated pancreata used for single-cell sequencing. **h** t-SNE visualization of endocrine cells in *Fev*-lineage-traced E14.5 mouse pancreata (*n* = 3). **i** Expression of major markers of endocrine cell types. Color indicates level of expression, except for the *eGFP* plot, which indicates presence or absence of *eGFP* counts

As the in vivo lineage tracing data had revealed that the Fev^Hi^ population is derived from the Ngn3+ population, we hypothesized that the Fev^Hi^ population could then function as a progenitor for the endocrine populations of the developing pancreas. We utilized a *Fev-Cre; ROSA26*^*mTmG*^ lineage tracing strategy to label *Fev*-expressing cells and their progeny. We found that 100% of alpha, beta, and delta cells, 90.1% of gamma cells, and 23.2% of epsilon cells were lineage-traced in E14.5 pancreas (Fig. 6a–e). These proportions of lineage labeling held true later in development (E17.5) and in adulthood (6 weeks) (Supplementary Fig. 6 and 7). Epsilon cells are rare in the adult pancreas^33^ and still exhibited only partial lineage tracing in E17.5 pancreas (47.8% traced) (Supplementary Fig. 6e). These results demonstrate that the majority of endocrine cells pass through a *Fev*-expressing stage during development.

We next combined this lineage tracing approach with single-cell RNA sequencing to identify transcriptional regulators of endocrine differentiation. FACS sorting was used to enrich for *Fev*-expressing cells and their progeny (membrane GFP+) from *Fev-Cre*; *ROSA26*^*mTmG*^ pancreata at E14.5 (Fig. 6f, g). All expected endocrine populations were identified in the resulting single-cell dataset (Fig. 6h, i). In addition, we found that *eGFP* reads mapped to all endocrine populations except the Ngn3+ population (Fig. 6i), further confirming that *Fev* expression turns on after *Ngn3*.

**Fig. 7:**
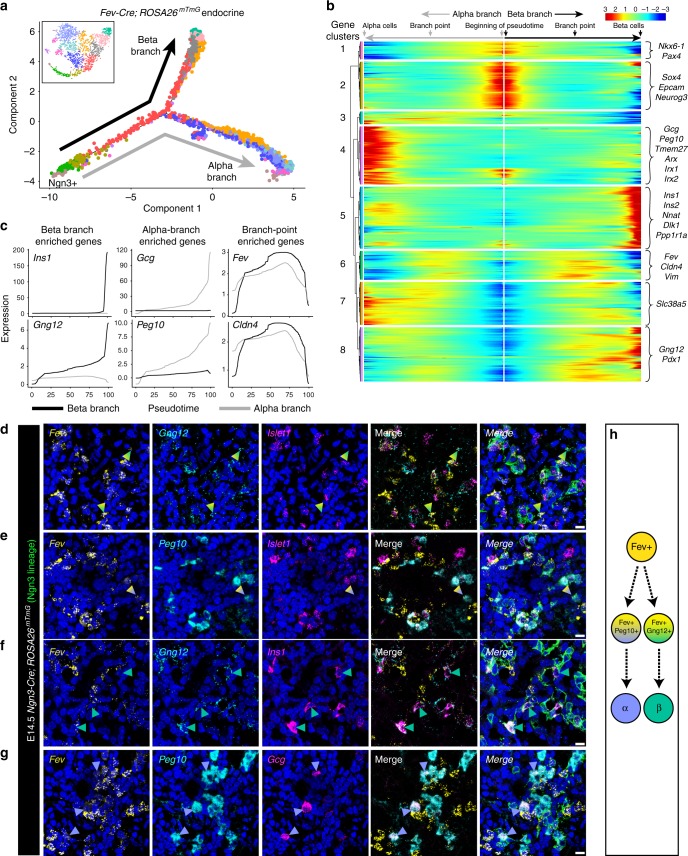
Identification of candidate regulators of beta and alpha cell fate decisions. **a** Pseudotime ordering of the endocrine cells at E14.5 depicted in Fig. 6h yields a bifurcated tree in which the two main branches terminate in cells that highly express *Ins1* (beta cell branch) or *Gcg* (alpha cell branch). **b** Heatmap depicting the expression of genes along each branch, in pseudotime. An independent expression pattern is calculated across the entire pseudotime trajectory for each branch. Therefore, the portion of the trajectory before the branch point is displayed for each branch separately. Genes are clustered based on expression pattern across pseudotime; selected genes with differential expression along the branches are highlighted to the right. **c** Gene expression plots depicting the kinetic trends along each branch. **d**–**e** Multiplexed fluorescent ISH for *Fev*, *Gng12*, and *Islet1* (**d**) or *Fev, Peg10*, and *Islet1* (**e**) in lineage-traced E14.5 *Ngn3-Cre; ROSA26*^*mTmG*^ pancreas. Arrowheads identify lineage-traced Fev+/Islet1− cells with *Gng12* (**d**, teal gradient arrowheads) or *Peg10* (**e**, indigo gradient arrowheads) expression. **f** Multiplexed fluorescent ISH for *Fev*, *Gng12*, and *Ins1*. Teal arrowheads identify lineage-traced Ins1+ beta cells that express *Gng12*. **g** Multiplexed fluorescent ISH for *Fev*, *Peg10*, and *Gcg*. Indigo arrowheads identify lineage-traced Gcg+ alpha cells that express *Peg10*. **h** Model for Fev^Hi^ (yellow) cell differentiation into distinct alpha or beta cells. *Peg10* and *Gng12* expression in Fev^Hi^ cells may represent progenitors pre-fated towards the alpha and beta lineages, respectively, during endocrine lineage allocation. **d**–**g** Scale bars represent 10 µm. Blue staining represents DAPI-labeled nuclei. Colors of arrowheads match colors of cells represented in **h**

We next set out to model the lineage relationships among the endocrine cells and identify transcriptional regulators of differentiation. Pseudotime ordering identified a trajectory that began with Ngn3+ cells, transitioned into Fev+ cells, and then split into two main branches (Fig. 7a; see similar branching pattern in analysis of our first v1 dataset, Supplementary Fig. 8a). The termini of the branches were populated by differentiated beta and alpha cells, suggesting that the branches represent a transition from a progenitor to fully differentiated hormone+ cell (Fig. 7a).

We next used Monocle’s branched expression analysis modeling (BEAM) to identify the genes that distinguish the paths along the two branches to either alpha or beta cells. We found gene clusters that were upregulated along different segments of the pseudotime trajectory (Fig. 7b and Supplementary Data 15) and performed pathway analysis to identify pathways enriched at each stage of pseudotime (Supplementary Fig. 8c and Supplementary Data 16). Genes upregulated at the beginning of pseudotime in gene cluster 2, included early markers of endocrine differentiation, such as *Sox4* and *Ngn3* (Fig. 7b). *Fev* was in gene cluster 6 and increased in both branches before ultimately decreasing in expression at the branch termini (Fig. 7b, c). Gene cluster 6 also included other genes expressed within the Fev^Hi^ population, including *Cldn4*, *Vim*, and *Chgb* (Fig. 7b, c and Supplementary Fig. 8b). We found branch-specific clusters that included known markers of beta (*Ins1*) and alpha (*Gcg*) cells and known differentiation regulators of alpha (*Arx*, *Pou3f4, Irx1, Slc38a5*, and *Tmem27*) and beta (*Pdx1, Pak3*, and *Nkx6-1*) cells (Fig. 7c and Supplementary Fig. 8b)^2,42–45^. These clusters also contained genes that were enriched in either the alpha or beta branch but were expressed before acquisition of hormone expression (Supplementary Fig. 8b). Within the alpha cell branch, *Peg10*, *Smarca1*, *Auts2*, and *Wnk3* increased in expression before upregulation of *Gcg* occurred (Supplementary Fig. 8b). *Peg10* and *Auts2* have roles in differentiation^46,47^ and migration^48^, but a role in endocrine differentiation has not been described. As a regulator of chromatin states and an adult human alpha cell marker^49^, *Smarca1* may be involved in the epigenetic regulation of alpha cell differentiation. Within the beta cell branch, *Gng12*, *Tssc4*, *Ece1, Tmem108*, *Wipi1*, and *Papss2* increased in expression before upregulation of *Ins1* (Supplementary Fig. 8b). To our knowledge, a role in endocrine lineage decisions have not been described for these beta branch-specific genes. We found a similar endocrine differentiation trajectory by an orthogonal method that uses force-directed layouts to visualize gene topologies and infer lineage relationships within single-cell data^50,51^ (Supplementary Fig. 8d). We hypothesize that the genes identified by the analysis above may represent regulators of the differentiation of an endocrine progenitor to a fully differentiated hormone-expressing cell.

To validate our pseudotime results, we performed ISH for markers that defined each branch of the trajectory. First, we confirmed the expression of *Peg10* and *Gng12* within the Fev^Hi^ population (Fig. 7d, e, indigo and teal gradient arrows), validating the expression of these genes in a stage before hormone acquisition. We also validated the enrichment of *Peg10* and *Gng12* in alpha and beta cells, respectively (Fig. 7f, g, solid indigo and teal arrows). First, 95.8% of beta cells expressed *Gng12* (*n* = 46 cells, 6 pancreata), while 30.5% expressed *Peg10* (*n* = 71 cells, 7 pancreata) (Fig. 7f and Supplementary Fig. 9a). Additionally, 100% of alpha cells expressed *Peg10* (*n* = 31 cells, 6 pancreata), while only 5.4% expressed *Gng12* (*n* = 32 cells, 4 pancreata) (Fig. 7g and Supplementary Fig. 9b). The lineage relationships generated by pseudotime ordering, combined with the validation in vivo, lead us to hypothesize that the Fev+/Peg10+ cells are fated toward an alpha cell identity and Fev+/Gng12+ cells toward a beta cell identity (Fig. 7h). These results suggest that lineage allocation of endocrine progenitors toward alpha or beta cell fates may occur after the onset of *Fev* expression.

## Discussion

The mesenchyme is critical for epithelial specification and proliferation throughout pancreatic development^52–54^, yet the individual cell types responsible for these processes remain unidentified. Our single-cell dataset has enabled the identification of multiple mesenchymal subpopulations and gene candidates for regulating epithelial–mesenchymal interactions. Secreted factors, such as mesothelial-derived Fgf9, may play a similar role in the pancreas as in the lung, where it regulates mesenchymal cell proliferation and vascular formation^55^. Additionally, secretion of Wnt antagonists by cluster 5 may regulate Wnt signaling in the developing pancreas, influencing processes such as epithelial specification, expansion, and exocrine development^56^. Future work can focus on uncovering the functions of these individual mesenchymal populations in the development, physiology, and pathology of the pancreas.

With the cell types of the mesenchyme now enumerated and their markers identified, we can begin to elucidate the maturation and lineage relationships of the pancreatic mesenchymal compartment. Our time-course data have provided evidence of maturation within the mesothelial population. Genes such as *Pitx2*, *kallikren 13* (*Klk13)* and 8 (*Klk8*), were differentially expressed in younger, E12.5 mesothelial cells. *Pitx2* regulates differentiation in multiple systems^24,57,58^, and the kallikren family are serine proteases involved in the degradation of extracellular matrix and adhesion molecules^59^. Expression of these genes suggests that the E12.5 mesothelial population may be primed for migration and differentiation. In contrast, the E17.5 mesothelial population expressed genes related to barrier or immune function, such as *dermokine* (*Dmkn*)^60,61^, *bone marrow stromal antigen 2* (*Bst2*), and *retinoic acid receptor responder 2* (*Rarres2*)^62^. These results suggest stage-dependent roles for the mesothelium throughout development.

The different roles for the mesothelium across time are also evident from our pseudotime analysis, which proposes that the mesothelium serves as a progenitor for other mesenchymal cell types during development. The mesothelium is a critical mesenchymal progenitor population in other organs, such as the heart, intestine, lung, and liver^16–19^. Our data suggest that mesothelial progenitor activity occurs at E12.5 or earlier during pancreatic development, consistent with other organ systems^11,16,18^. Indeed, a recent study identified that parietal mesothelial cells can function as progenitor cells prior to pancreatic specification^63^. The transcriptomic information obtained by this study will allow the development of tools to target individual populations within the mesenchyme and perform lineage tracing, ablation, and expression studies in vivo. Furthermore, this developmental dataset can be compared to mesenchymal population dynamics during adult disease progression, where aberrant recapitulation of developmental pathways can lead to disease states in the pancreas^64,65^. Thus, this dataset is a broad resource for the implementation of future studies in pancreatic mesenchymal biology.

Within the epithelial compartment, our identification of a Fev^Hi^ endocrine progenitor population provides increased resolution of endocrine differentiation. The relative timing of expression of canonical endocrine lineage genes can now be mapped onto these additional differentiation stages. Several lines of evidence suggest that the gene *Fev* may be a direct target of Ngn3: *Fev* is the transcription factor most strongly expressed in Ngn3+ endocrine progenitors^66^, and *Ngn3* knockout embryos do not express *Fev* in the developing pancreas^26^. Known target genes of *Ngn3*, such as *Pax4*^67^ and *Runx1t1*^68^, are expressed by the early-stage Fev+/Pax4+ population. Additionally, *Pax6* is upregulated within the Fev^Hi^ population. Although *Chga* and *Chgb* are often utilized as markers of differentiated endocrine lineages, we found that both are expressed in the Fev^Hi^ population prior to hormone acquisition. This result is consistent with previous work that identified Chga+, hormone– cells in rodent pancreatic development^69^. The Fev^Hi^ cell stage likely represents the cell stage during endocrine differentiation preceding specialized hormone production and may now serve as a cellular landmark for understanding endocrine lineage gene-expression dynamics.

The gene *Fev* has been previously studied mainly in serotonergic neurons, where it is a master transcriptional regulator required for cellular differentiation, maturation, and serotonin synthesis^31^. Fev switches transcriptional targets from differentiation genes during development to maturation genes postnatally in serotonergic neurons^70^. In an insulinoma cell line, Fev directly binds to the regulatory regions of serotonergic genes, such as *Tph1*, *Tph2*, *Ddc*, *Slc18a2*, and *Slc6a4*, as well as the *Ins1* promoter itself^26^. Future ChIP-seq studies of embryonic pancreas will globally identify direct targets of Fev and Fev-regulated transcriptional networks in developing endocrine cells.

Using genetic lineage tracing in vivo, we have demonstrated that the majority of endocrine cells in the developing pancreas transit through a *Fev*-expressing stage, and that Fev-lineage cells contribute not only to embryonic, but also to adult pancreatic endocrine cells. The fraction of epsilon cells not derived from a Fev-lineage may represent the subset of Ghrl+ cells previously reported to give rise to the ductal and exocrine lineages^33^. As all adult gamma cells are Fev-lineage labeled, the small subset of gamma cells not lineage traced during pancreatic development may represent those that do not persist in the adult pancreas.

Further highlighting the relevance of Fev^Hi^ progenitors during pancreatic development, our pseudotime analysis revealed that *Fev*-expressing cells may be pre-specified towards an alpha or beta cell fate. As expected, we found expression of *Ins1* and *Gcg* at the termini of the beta and alpha branches, and upregulation of *Pdx1* and *Arx*, which are known regulators of endocrine cell fate decisions, earlier in pseudotime. In addition, our pseudotime analysis identified genes enriched along the alpha or beta branch and expressed prior to upregulation of hormones. These genes warrant further study as potential regulators of the acquisition of alpha or beta cell identity.

For the eventual application of this knowledge to human therapeutics, the findings in the murine model must be validated in human pancreatic development. Our staining of human fetal pancreas identified the analogous FEV^Hi^ population, consistent with our findings in murine pancreata. Directed differentiation of hESCs toward endocrine cell fates will provide a platform for modeling and manipulating the putative lineage regulators found in this study. Indeed, we have identified a FEV+ population within hESC-derived endocrine progenitor cells. Deeper knowledge of these lineage decisions may substantially improve directed differentiation efforts to efficiently generate functional beta cells for cellular replacement therapy for people with diabetes. This study highlights the power of combining single-cell transcriptomic information with in vivo lineage tracing to reconstruct developmental trajectories within cellular compartments. Discovery of populations and their lineage relationships will promote identification of the mechanisms that drive lineage decisions and commitment.

## Methods

### Mice

All mouse procedures were approved by the University of California, San Francisco (UCSF) Institutional Animal Care and Use Committee. Mice were housed in a 12-h light-dark cycle in a controlled temperature climate. Noon of the day of vaginal plug was considered embryonic day 0.5.

Timed-pregnant Swiss Webster mice were obtained from Charles River Laboratories. *Ngn3-Cre*^71^ (a gift from Dr. Matthias Hebrok), *Fev-Cre*^72^ (The Jackson Laboratory 012712), and *ROSA26*^*mTmG* 34^ (the Jackson Laboratory 007676) mice were maintained on a C57BL/6J background. The *Cre* transgene was genotyped using the following primers: GGGCGGCATGGTGCAAGTT and CGGTGCTAACCAGCGTTTTC.

### Human tissue procurement and isolation

Human fetal pancreata were harvested from post-mortem fetuses at 23 weeks of gestation with permission from the ethical committee of UCSF. Tissue was fixed in 4% paraformaldehyde overnight at 4 °C. After three washes in 1X phosphate-buffered saline (PBS), tissue was either cryopreserved in 30% sucrose solution at 4 °C overnight and embedded in optimal cutting temperature (OCT) compound, or placed in 40% ethanol then 70% ethanol before paraffin embedding. Sections measuring 8 µm were cut on the cryostat or microtome. In situ hybridization and immunofluorescence were then performed as described below.

Adult human islets were isolated from cadaveric donor tissue by the UCSF Islet Production Core with permission from the UCSF ethical committee. Consented cadaver donor pancreata were provided by the nationally recognized organization UNOS via local organ procurement agencies. The identifiers were maintained at the source only, and the investigators received de-identified specimens.

Informed consent was obtained for all human (fetal and adult) tissue collection, and protocols were approved by the Human Research Protection Program Committee on Human Research of UCSF.

### Embryonic stem cell culture and differentiation

The hESC line HUES8 was obtained from Harvard University and used for the generation of hESC-derived BLCs. Pluripotent HUES8 cells were maintained as spherical clusters in suspension in mTeSR-1 (StemCell Technologies) in 500 mL spinner flasks (Corning, VWR) on a magnetic stir plate (Dura-Mag) within a 37 °C incubator at 5% CO_2_, 100% humidity, and a rotation rate of 70 rpm. Cells were screened for mycoplasma contamination using the MycoProbe Mycoplasma Detection Kit (R&D Systems), according to the manufacturer’s instructions.

hESC-derived endocrine progenitor cells were generated as previously described^35^. In brief, HUES8 cells were seeded into a spinner flask at a concentration of 8 × 10^5^ cells/mL in mTeSR-1 media with 10 μM Rock inhibitor Y27632 (StemCell Technologies) to allow formation of spherical clusters. Differentiation was initiated 72 h later. Differentiation was achieved in a step-wise fashion using the following growth factors and/or small molecules: definitive endoderm (Stage 1) (1 day of 100 ng/mL Activin A (R&D Systems) and 14 μg/mL of CHIR99021 (Stemgent); 2 days of 100 ng/mL Activin A); gut tube endoderm (Stage 2) (3 days of 50 ng/mL KGF (Peprotech)); early pancreatic progenitors (Stage 3) (1 day of 200 nM LDN193189 (Fisher Scientific), 50 ng/mL KGF, 0.25 μM Sant-1 (Sigma), 2 μM Retinoic Acid (Sigma), 500 nM PdbU (EMD Biosciences); 1 day of 50 ng/mL KGF, 0.25 μM Sant-1, 2 μM Retinoic Acid, 500 nM PdbU); later pancreatic progenitors (Stage 4) (5 days of 50 ng/mL KGF, 0.25 μM Sant-1, 0.1 μM Retinoic Acid); endocrine progenitors (Stage 5) (4 days of 0.25 μM Sant-1, 0.1 μM Retinoic Acid, 1 μM XXI (EMD Millipore), 10 μM Alk5i (Axxora), 1 μM T3 (EMD Biosciences), 20 ng/mL Betacellulin (Fisher Scientific); 3 days of 25 nM Retinoic Acid, 1 μM XXI, 10 μM Alk5i, 1 μM T3, 20 ng/mL Betacellulin); BLCs (Stage 6) (6 days of 10 μM Alk5i; 1 μM T3). Successful differentiation was assessed at Stages 1, 3, 4, 5, and 6 via immunofluorescence or FACS for stage-specific marker genes.

To measure the expression of *FEV* at various stages of human endocrine differentiation, aliquots of clusters were removed from the flask and analyzed at several timepoints: after 5 days in Stage 5 (“mid-stage endocrine progenitors”), after 7 days in Stage 5 (“late-stage endocrine progenitors”), and after 5 days at the BLC stage. As a comparator, pluripotent, undifferentiated hESCs in mTeSR-1, as well as human adult islets, were also analyzed for *FEV* expression.

### Immunofluorescence

Embryonic mouse pancreata were dissected in cold 1X PBS and fixed in zinc-buffered formalin (Anatech LTD) at room temperature (RT) for 30–90 min or overnight at 4 °C. After three washes in 1X PBS, tissue was processed for either cryopreservation or paraffin embedding. Cryopreserved pancreata were placed in 30% sucrose solution at 4 °C overnight before embedding in OCT. Paraffin-embedded pancreata were placed in 40% ethanol and 70% ethanol before paraffin tissue processing. Sections measuring 8 µm sections were cut on the cryostat or microtome. For immunofluorescence on paraffin sections, slides were baked at 55 °C for 30 min, deparaffinized in xylene, and rehydrated in decreasing concentrations of ethanol. Heat-mediated antigen retrieval was performed using Antigen Retrieval Citra Solution (Biogenex Laboratories). Tissue sections were blocked in 5% normal donkey serum (NDS; Rockland Immunochemicals) and Mouse-on-Mouse IgG blocking reagent (Vector Laboratories) when appropriate in 0.2% Triton X-100 in PBS (PBT) for 1 h and then stained overnight at 4 °C using the following primary antibodies: Acta2 (1:200, Abcam ab21027), Cav1 (1:200, Abcam ab2910), Chromogranin A (1:100, Abcam ab15160), E-cadherin (1:200, BD Transduction Lab 610182), Glucagon (1:100, Abcam ab82270), Insulin (1:50, DAKO A0564), Vimentin (1:200, Abcam ab92547), and Wt1 (1:100, Abcam ab89901). All antibodies have been validated by the manufacturer. The next day, sections were washed three times in 0.1% Tween 20 in 1X PBS and then incubated with species-specific Alexa Fluor 488-, 594-, or 647-conjugated secondary antibodies (1:500, Jackson ImmunoResearch) and DAPI in 5% NDS in 0.2% PBT for 1 h at RT. Sections were washed three times in 0.1% Tween 20 in 1X PBS, rinsed in 1X PBS, and then mounted in Fluoromount-G mounting medium (SouthernBiotech). Slides were stored at 4 °C.

For immunofluorescence on cryosections, slides were removed from −80 °C storage and allowed to reach RT. Sections were rinsed in 1X PBS three times and permeabilized in 0.5% PBT for 10 min at RT. Tissue sections were blocked in 5% NDS and, if needed, Mouse-on-Mouse IgG blocking reagent in 0.1% PBT for 1 h and then stained overnight at 4 °C using the following primary antibodies: Epcam (1:200, BD Transduction Lab 552370), Glucagon (1:2000, Millipore 4031-01F), Insulin (1:250, DAKO A0564), Somatostatin (1:500, Santa Cruz Biotechnology) sc-7819, Ghrelin (1:1500, Santa Cruz Biotechnology sc-10368), Pancreatic Polypeptide (PP; 1:250, Abcam ab77192), and Vimentin (1:200, Abcam ab92547). All antibodies have been validated by manufacturer. Sections were washed the next day three times in 1X PBS and then incubated with species-specific Alexa Fluor 488-, 555-, 594-, or 647-conjugated secondary antibodies and DAPI in 5% NDS in 0.1% PBT for 1 h at RT. Sections were washed three times in 1X PBS and mounted in Fluoromount-G mounting medium. Slides were stored at 4 °C.

Images were captured on a Zeiss Apotome Widefield microscope with optical sectioning capabilities or Leica confocal laser scanning SP8 microscope. Maximum intensity z-projections were then prepared using ImageJ, where brightness, contrast, and pseudo-coloring adjustments were applied equally across all images in a given series.

### In situ hybridization

In situ hybridization was performed on 8 µm sections using RNAscope technology (Advanced Cell Diagnostics)^73^ according to the manufacturer’s instructions. In situ probes against mouse *Ngn3* (422409-C2), *Fev* (413241-C3), *Isl1* (451931), *Ins1* (414661-C4), *Gcg* (400601), *Sst* (404631-C3), *Ghrl* (415301-C2), *Ppy* (482701), *Peg10* (512921-C4), *Gng12* (462521-C2), *Nnat* (432631-C2)*, Barx1* (414681)*, Pitx2* (412841-C2)*, Stmn2* (498391-C3)*, Msln* (443241) and human *NGN3* (505791-C4), *FEV* (471421-C3), and *ISL1* (478591-C2) were used in combination with the RNAscope Multiplex Fluorescent Reagent Kit v2 for target detection. Following signal amplification of the target probes, sections were washed in 1X PBS three times and blocked in 5% NDS in 0.1% PBT for 1 h at RT. Tissue sections were then stained with primary and secondary antibodies as described above in the “immunofluorescence” section.

For in situ hybridization of hESC-derived clusters, cells were fixed with 4% PFA for 15 min at RT, washed with PBS, and cryoprotected in 30% sucrose overnight. The next day, clusters were embedded in a small sphere of 1.5% low-melting temperature agarose; these were again cryoprotected in 30% sucrose overnight. The following day, the agarose spheres were soaked in OCT and frozen in a dry ice bath. In situ hybridization was then performed on 8 µm sections using human *NGN3*, *FEV*, and *ISL1* RNAscope probes.

### Quantification of cell proportions

Quantification of pancreata was performed by manual counting using ImageJ software. Cell populations present at less than 1% in *Ngn3*-lineage-traced E14.5 replicates were deemed artifact and excluded from further analysis.

### Quantitative RT-PCR

hESCs from various stages of directed differentiation were collected and RNA was extracted with the RNeasy Mini Kit (Qiagen). Reverse transcription was performed with the Clontech RT-PCR kit. RT-PCR was run on a 7900HT Fast RT-PCR instrument (Applied Biosystems) with Taqman probes for *FEV* (assay ID: Hs00232733_m1) and *GAPDH* (assay ID: Hs02758991_g1) in triplicate. Data were normalized to *GAPDH*. Error bars represent standard deviation.

### Dissociation and FACS of embryonic pancreas

Embryonic mouse pancreata were dissected and placed in 1X PBS on ice, then dissociated into single cells using TrypLE Express dissociation reagent (Thermo Fisher) at 37 °C with pipet trituration at 5-min intervals during incubation. For v1 datasets, E12.5 pancreata were dissociated for 10 min, E14.5 pancreata for 15 min, and E17.5 pancreata for 30 min. For batch 1, we pooled 14 E14.5 pancreata from one litter. For batch 2, which was collected on a different day, we pooled tissue from each timepoint separately: 18 E12.5 pancreata from two litters, 11 E14.5 pancreata from one litter, and 8 E17.5 pancreata from one litter. Dissociations were neutralized with FACS buffer (10% FBS+ 2 mM EDTA in phenol-red free HBSS). Dissociated cells were passed through a 30 µm cell strainer and stained with Sytox live/dead stain (Thermo Fisher). Stained cells were washed twice in FACS buffer and then sorted using a BD FACS Aria II. After size selection to remove doublets, all live cells were collected.

For v2 10X datasets, we pooled tissue from each time point separately, each performed on a different day: 14 E12.5 pancreata from one litter, 13 E14.5 pancreata from one litter, and 13 E17.5 pancreata from one litter. For the E14.5 *Fev-Cre; ROSA26*^*mTmG*^ 10X sample, we pooled three pancreata from one litter. Dissociations were performed as described above. Cells undergoing a CD140a negative selection were stained with CD140a-APC (1:50; eBiosciences, cat. 17-1401-81; validated by manufacturer). Stained cells were washed twice in FACS buffer and then sorted using a BD FACS Aria II. After size selection to remove doublets, all live CD140a− cells were collected. For the E14.5 *Fev-Cre; mTmG* pancreata, live GFP+ cells and GFP−/TdTomato+ cells were collected. All 4000 GFP+ (*Fev*-lineage traced) cells were loaded onto the 10X Genomics platform, supplemented with an additional 21,000 TdTomato+/GFP− (non-lineage traced).

### Single-cell capture and sequencing

To capture individual cells, we utilized the Chromium Single Cell 3' Reagent Version 1 Kit (10X Genomics)^74^. For batch 1, 12,800 cells from E14.5 pancreata were loaded into one well of the 10X chip, while for batch 2, 18,000 cells per time point were each loaded into their own respective wells to produce Gel Bead-in-Emulsions (GEMs). GEMs underwent reverse transcription to barcode RNA before cleanup and cDNA amplification. Libraries were prepared with the Chromium Single Cell 3' Reagent Version 1 Kit. Each sample was sequenced on 2 (Batch 1) or 1 (Batch 2) lanes of the HiSeq2500 (Illumina) in Rapid Run Mode with paired-end sequencing parameters: Read1, 98 cycles; Index1, 14 cycles; Index2, 8 cycles; and Read2, 10 cycles.

The CD140a-depleted E12.5, E14.5, and E17.5 datasets and *Fev-Cre; ROSA26*^*mTmG*^ dataset in Figs. 5 and 7 were generated with Chromium Single Cell 3' Reagent Version 2 kits (10X Genomics). In total, 27,000 cells were loaded onto their respective wells and underwent the same processing as the Version 1 kits, according to manufacturer instructions for Version 2 kits. Libraries were sequenced on the NovaSeq (Illumina) with the same sequencing parameters as above.

### Single-cell analysis

For the v1 datasets, we utilized CellRanger v1.1.0 software for v1 datasets and v2.1.0 for v2 datasets with default settings for de-multiplexing, aligning reads to the mouse genome (10X Genomics pre-built mm10 reference genome) with STAR^75^ and counting unique molecular identifiers (UMIs) to build transcriptomic profiles of individual cells. For the v1 datasets, gene-barcode matrices were analyzed with the R package Seurat v1.4, using the online tutorial as a guide^7,76^. We first performed a filtering step, retaining only the cells that expressed a minimum of 200 genes and only the genes that were expressed in at least 3 cells. A large number of cells did not meet this threshold in the E17.5 time point and were determined to be red blood cells by the high expression of hemoglobin genes. Variable genes were determined by mean-variance relationship to identify highly expressed and variable genes with the Seurat function MeanVarPlot with default settings. UMI counts were log-normalized, and linear regression was performed with RegressOut to account for differences in the number of UMIs between cells. PCA was then utilized to determine sources of variability in the dataset with PCAfast. Significant PCs were determined based on the Scree plot and utilized for Seurat’s graph-based clustering algorithm (function FindClusters) with default parameters, except for the resolution parameter. To vary cluster numbers, the resolution parameter in FindClusters was adjusted from 0.6 to 3.0, and resulting clusters analyzed as follows. Clusters were visualized by t-SNE with Seurat’s RunTSNE function using default settings^77^. Differentially expressed genes were determined with the FindAllMarkers function, which uses a bimodal likelihood ratio test^8^. We confirmed differential gene-expression analysis with the Wilcoxon rank sum test and Model-based Analysis of Single-cell Transcriptomics (MAST)^9^ utilizing Seurat v2’s FindMarkers function with default settings. These tests calculate adjusted *p* values for multiple comparisons. To determine the final number of clusters, clusters were required to have at least nine significantly (*p* < 0.05) differentially expressed genes with a twofold difference in expression in comparison to all other clusters. Clusters were manually curated for differential gene expression, and those that did not meet this threshold were manually merged with the nearest cluster based on the phylogenetic tree from Seurat’s BuildClusterTree. In some cases, clusters met the nine-gene threshold but appeared to have very similar differentially expressed genes to another cluster. This is likely a result of the comparison of individual clusters against all other clusters in determining differentially expressed genes. In these cases, a pairwise comparison between the two clusters was performed and the same nine-gene threshold applied. An exception to the nine-gene threshold was made to annotate the proliferating population in early stages of the cell cycle within the E14.5 mesenchymal analysis (Fig. 4, cluster 8). Additionally, cluster 10 in the E14.5 mesenchymal dataset did not meet the nine-gene threshold. Rather, clusters 1–9 had distinct transcriptomic signatures (with at least nine differentially expressed genes) that distinguished them from cluster 10. Lists of at least two-fold differentially expressed genes for individual analyses are provided in Supplementary Data 2.

For v2 datasets, Seurat v2.2 and v2.3 was utilizing to perform the analysis. Cells with fewer than 200 genes and genes expressed in fewer than 3 cells were removed, as above. UMI counts were normalized with NormalizeData using default settings. Variable genes were determined with FindVariableGenes, using the following cut-offs suggested by the online tutorial (x.low.cutoff = 0.0125, x.high.cutoff = 3, and y.cutoff = 0.5). Data were scaled and UMI counts regressed out with the ScaleData function. PCA was performed with RunPCA, and significant PCs determined based on the Scree plot. t-SNE analysis and clustering was performed as described above for the v1 datasets. For the E12.5 exocrine dataset, the ductal population did not meet the nine-gene threshold. All other populations within this dataset could be distinguished from the ductal population by at least nine differentially expressed genes, therefore we still annotated this cluster. Some of the clusters depicted for the *Fev-Cre; ROSA26*^*mTmG*^ dataset do not meet the nine-gene threshold. We chose to visualize these clusters in order to better illustrate their placement along the pseudotime trajectory.

### Custom genome build

The custom genome for alignment of reads to eGFP and TdTomato sequences from the mTmG mouse line was created according to instructions provided by 10X Genomics reference support (https://support.10xgenomics.com/single-cell-gene-expression/software/pipelines/latest/advanced/references). eGFP and TdTomato sequences were concatenated to the mm10-2.1.0 reference genome (FASTA file) provided by 10× Genomics. eGFP and TdTomato annotations were then concatenated to the mm10 annotations (GTF file) provided by 10X Genomics. The cellranger mkref command was then utilized with the genome and annotations with eGFP and TdTomato, as described in the above link.

### Pathway analysis

Pathway analysis and calculation of associated *p* values were performed using the ConsensusPathDB overrepresentation analysis for pathway-based sets category (http://cpdb.molgen.mpg.de)^78^.

### Aggregating E17.5 v2 datasets

E17.5 technical replicates from the v2 dataset were aggregated with Cellranger v2.1, utilizing the aggr function with default settings. The aggregated dataset was used for analysis and merging with the E12.5 and E14.5 v2 datasets.

### Subclustering and merging datasets

Subclustering was performed by isolating clusters of interest with the Seurat function SubsetData and reanalyzing as described above (identification of variable gene, regression, and determination of significant PCs). Cells were classified as epithelial based on the expression of *E-cadherin* (*Cdh1*) and other known epithelial population markers. Cells that were *Cdh1−, Vim*+, and *Collagen3a1* (*Col3a1*)+ were classified as mesenchymal. Multiple batches were merged with the MergeSeurat function. The merged dataset was reanalyzed as above, with batch included as a latent variable in the RegressOut function. The v1 E14.5 batch 1 and batch 2 clusters were robust to the sampling differences between batches as evidenced by the contribution of cells from both batches to each cluster (Supplementary Fig. 2b). We found high correlation of cell type proportion between batches in all populations except the exocrine compartment (acinar and ductal) (Supplementary Fig. 2c), possibly due to technical challenges of pancreatic dissociation. Within each cluster, batch 1 cells correlated most highly with those of batch 2 contained in the same cluster, indicating proper cluster calling with the merged datasets (Supplementary Fig. 2d).

For v2 datasets (E12.5, E14.5, and E17.5), multiple canonical correlation analysis (multiCCA) from Seurat v2.3 was utilized to merge the epithelial datasets^39^. The top 1000 most highly variable genes that were variable in at least 2 datasets were used for the alignment, as recommended in the Seurat tutorial. The shared correlation strength of each CC was measured with Seurat’s MetageneBicorPlot, and those before the drop-off were used for alignment, analogous to the Scree plot in choosing significant PCs. We then aligned the datasets with AlignSubspace and ran an integrated t-SNE and clustering analysis, as outlined in the Seurat tutorial. Clusters were required to have 9 significantly differentially expressed genes as described above. Clusters with similar differentially expressed genes were verified with pairwise comparisons to the most related clusters (based on BuildClusterTree) and merged if they did not meet the pairwise nine-gene threshold. The Beta 2 cluster in the v2 endocrine merged timecourse data met the nine-gene threshold for two out of the three differential expression tests (Bimodal likelihood ratio and Wilcoxon rank sum tests), but had only eight differentially expressed genes for the MAST test.

Doublets were identified based on co-expression of two mutually exclusive genes, such as both mesenchymal and epithelial genes, and removed from further analysis. In the v2 datasets, rare cells (4 cells in E12.5 and 13 cells in E14.5 endocrine datasets) with high levels of hemoglobin gene expression were removed from the analysis.

### Downsampling analysis

To determine if the sequencing depth was sufficient for calling clusters, downsampling analysis was performed for the v1 E14.5 batch 1 dataset. Reads were randomly downsampled from the 10x Cellranger bam file output to a specified percentage, then grouped based on UMI to generate a count profile for each cell. The number of genes with greater than 0 counts was then calculated. UMI downsampling was performed with the SampleUMI function. A new Seurat object was created with the downsampled matrix and reanalyzed as above.

The number of UMIs/cell was downsampled from an average of 4600 UMIs/cell in the full dataset to 200 UMIs/cell, and the median number of genes/cell and clustering robustness was then calculated. Clustering robustness was determined as the percentage of cells within the same cluster, with clusters required to maintain at least nine genes with a twofold change in expression in comparison to all other clusters. Within this dataset, robust clustering was maintained all the way down to 500 UMIs/cell, when the percentage of cells in the same cluster began to climb, indicating collapsing of individual clusters. Both of these downsampling analyses indicate that sufficient sequencing depth was reached.

### Pseudotemporal ordering

We utilized Monocle 2.6.4^25^ to order cells in pseudotime based on their transcriptomic similarity. For v1 timecourse datasets, batch-corrected values and variable genes from Seurat analysis were used as input, utilizing the gaussianff expressionFamily, and clusters were projected onto the minimum spanning tree after ordering.

For the *Fev*-lineage-traced dataset, UMI counts and variable genes from the Seurat analysis were used as input, utilizing the negBinom expressionFamily. To find genes differentially expressed across the branch point in the trajectory, we used monocle’s internal BEAM analysis and selected genes with an FDR cutoff of 0.001. Gene expression patterns were plotted with plot_genes_branched_heatmap and plot_multiple_branches_pseudotime.

### Code availability

Seurat and monocle R objects used for analysis are available, along with scripts, at Figshare, 10.6084/m9.figshare.c.4158458.

Scripts are available at https://github.com/sneddonucsf/2018-Developmental-single-cell-RNA-sequencing.

## Electronic supplementary material

Supplementary Information

Description of Additional Supplementary Files

Supplementary Data 1

Supplementary Data 2

Supplementary Data 3

Supplementary Data 4

Supplementary Data 5

Supplementary Data 6

Supplementary Data 7

Supplementary Data 8

Supplementary Data 9

Supplementary Data 10

Supplementary Data 11

Supplementary Data 12

Supplementary Data 13

Supplementary Data 14

Supplementary Data 15

Supplementary Data 16

## Data Availability

RNA sequences for the single-cell RNA-sequencing analyses reported in this paper have been deposited in the GEO database under accession code GSE101099. The authors declare that all data supporting the findings of this study are available within the article and its supplementary information files or from the corresponding author upon reasonable request.
