# Supplementary material for: Lineage dynamics of murine pancreatic development at single-cell resolution
Source: Nat Commun. 2018 Sep 25;9:3922. doi: 10.1038/s41467-018-06176-3 (PMC6156586; doi:10.1038/s41467-018-06176-3)
Supplement: Supplementary file 1 — Supplementary Information [file 41467_2018_6176_MOESM1_ESM.pdf]

Lineage dynamics of murine pancreatic development at single-cell resolution

Byrnes et al.

Supplementary Figure 1

a

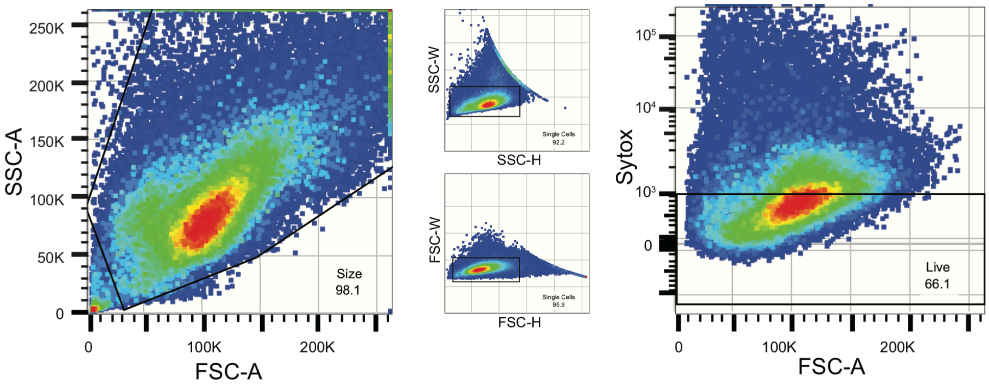

b

	E12.5 Batch 2	E14.5 Batch 1	E14.5 Batch 2	E17.5 Batch 2
**Cells Captured**	4,631	3,849	5,179	4,635
**Median reads/cell**	28,016	61,681	31,875	25,324
**Median UMIs/cell**	4,339	3,084	4,388	3,419
**Median genes/cell**	1,912	1,621	1,731	194
**Valid Barcodes**	75.70%	94.20%	92.50%	75.90%
**Mapped Reads to transcriptome**	65.40%	64.10%	66.70%	66.20%
**Sequencing Saturation**	58.60%	87.70%	71.20%	63.70%
**Fraction Reads in Cells**	90.10%	90.80%	93.00%	92.30%

c

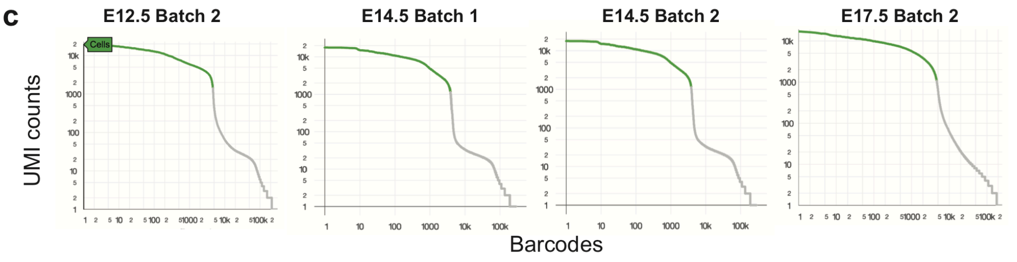

d

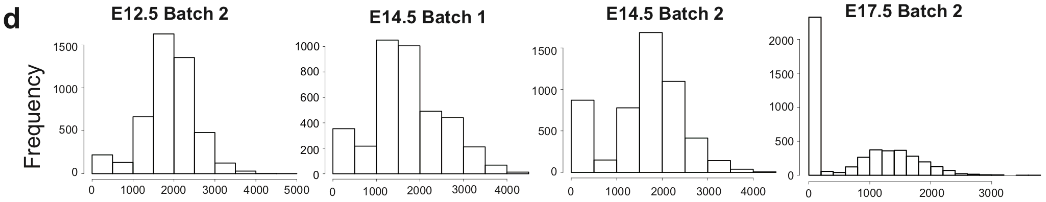

e

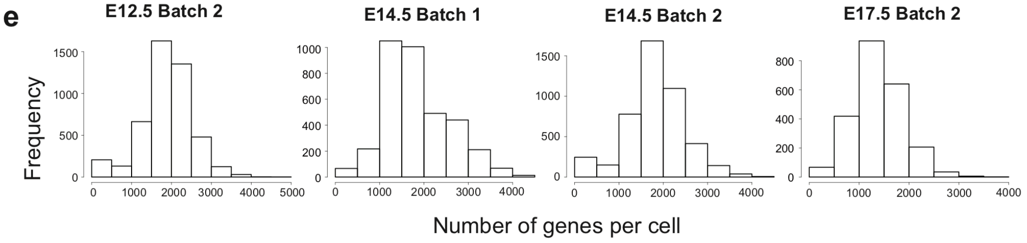

Supplementary Figure 1. Quality control for version 1 single-cell RNA-sequencing runs. (a) Representative FACS plot of single, live cells sorted from dissociated Swiss Webster embryonic pancreata and used for single-cell sequencing. (b) Quality control statistics for all single-cell sequencing runs prepared with the Chromium Single Cell 3' Reagent Version 1 Kit. The "valid barcodes" metric indicates the percentage of cells with barcodes that match a known barcode contained on a bead. "Mapped reads to transcriptome" refers to the percentage of reads that confidently map to a unique gene in the reference transcriptome. "Fraction Reads in Cells" is the percentage of reads that contain a cell-associated barcode. (c) Cellranger cell calls based on the number of UMIs. The dropoff indicates the threshold for the number of UMIs required for a barcode to be assigned to a cell. (d) Histogram of the number of genes per cell in all single-cell runs pre-filtering steps. (e) Histogram of the number of genes per cell in all single-cell runs post-filtering steps. E17.5 Batch 2 contained a large number of red blood cells, which expressed fewer than 200 genes, resulting in their removal during minimum gene threshold filtering (see Methods).

Supplementary Figure 2

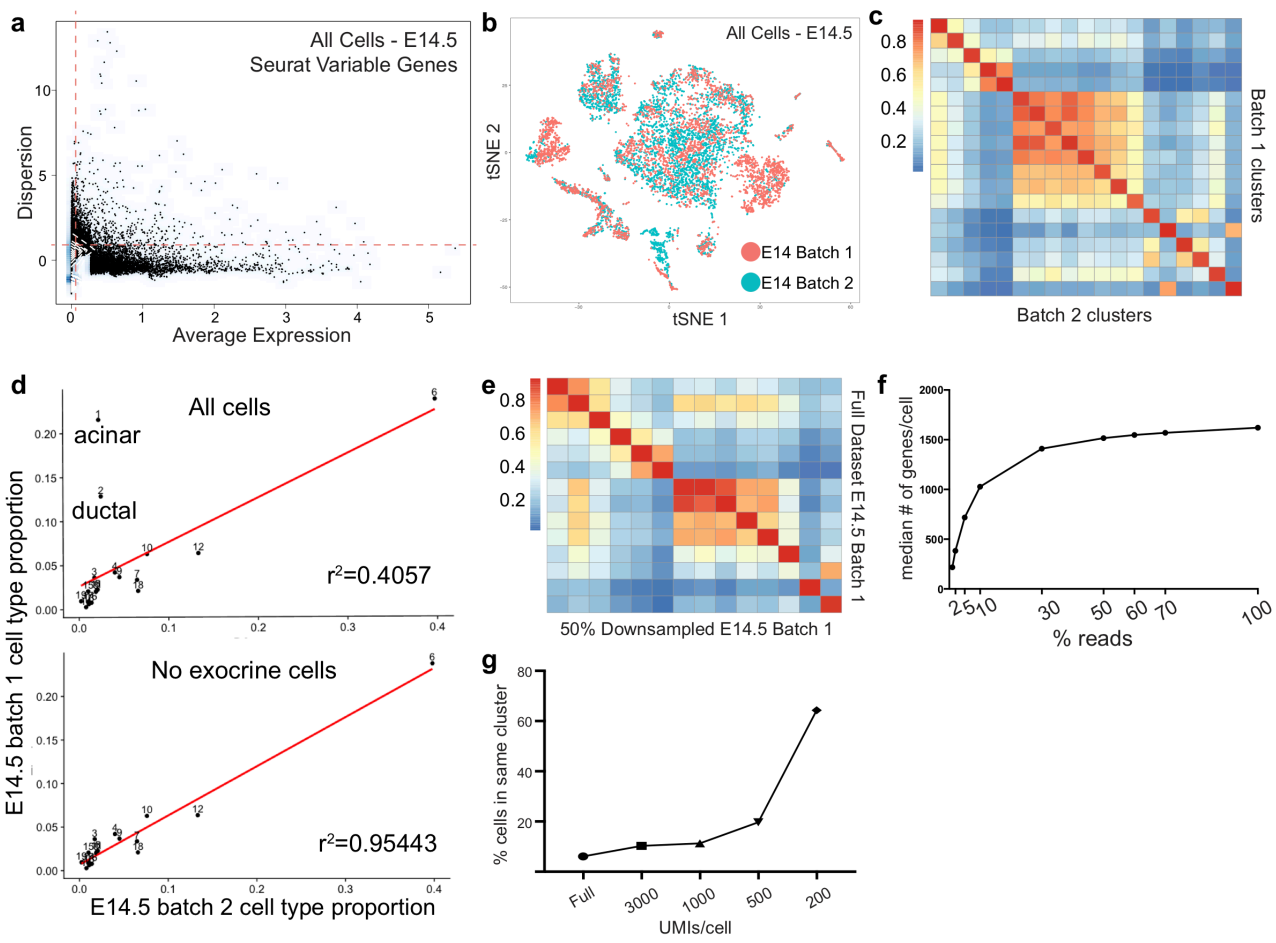

Supplementary Figure 2. Single-cell RNA-sequencing batch information from E14.5 pancreata.

(a) Selection of variable genes in the E14.5 v1 dataset (all cells) by Seurat's MeanVarPlot function. (b) t-SNE visualization of merged E14.5 batches, color-coded by batch. Batch 1 and 2 contribute to all clusters, reflecting a successful batch correction. (c) Pearson's correlation of E14.5 batch 1 cells with E14.5 batch 2 cells within each cluster based on average expression of variable genes. Batch 1 cells correlate most highly with batch 2 cells within the same cluster, indicating proper merging of the two batches. (d) Cell type proportions in E14.5 batch 1 and 2 with exocrine (acinar and ductal) clusters included (top panel) and excluded (bottom panel). All

cell types except the exocrine compartment show high correlation between the two batches. (e) Pearson’s correlation between clusters from the E14.5 batch 1 full dataset and those from the E14.5 batch 1 dataset downsampled to 50% of the reads, based on average expression of shared variable genes. (f) Maintenance of the number of median genes/cell after random downsampling of reads, indicating sufficient sequencing depth. (g) Maintenance of cluster structure after random downsampling of UMIs is reflected by the similar percentage of cells found within the same cluster with fewer UMIs.

Supplementary Figure 3

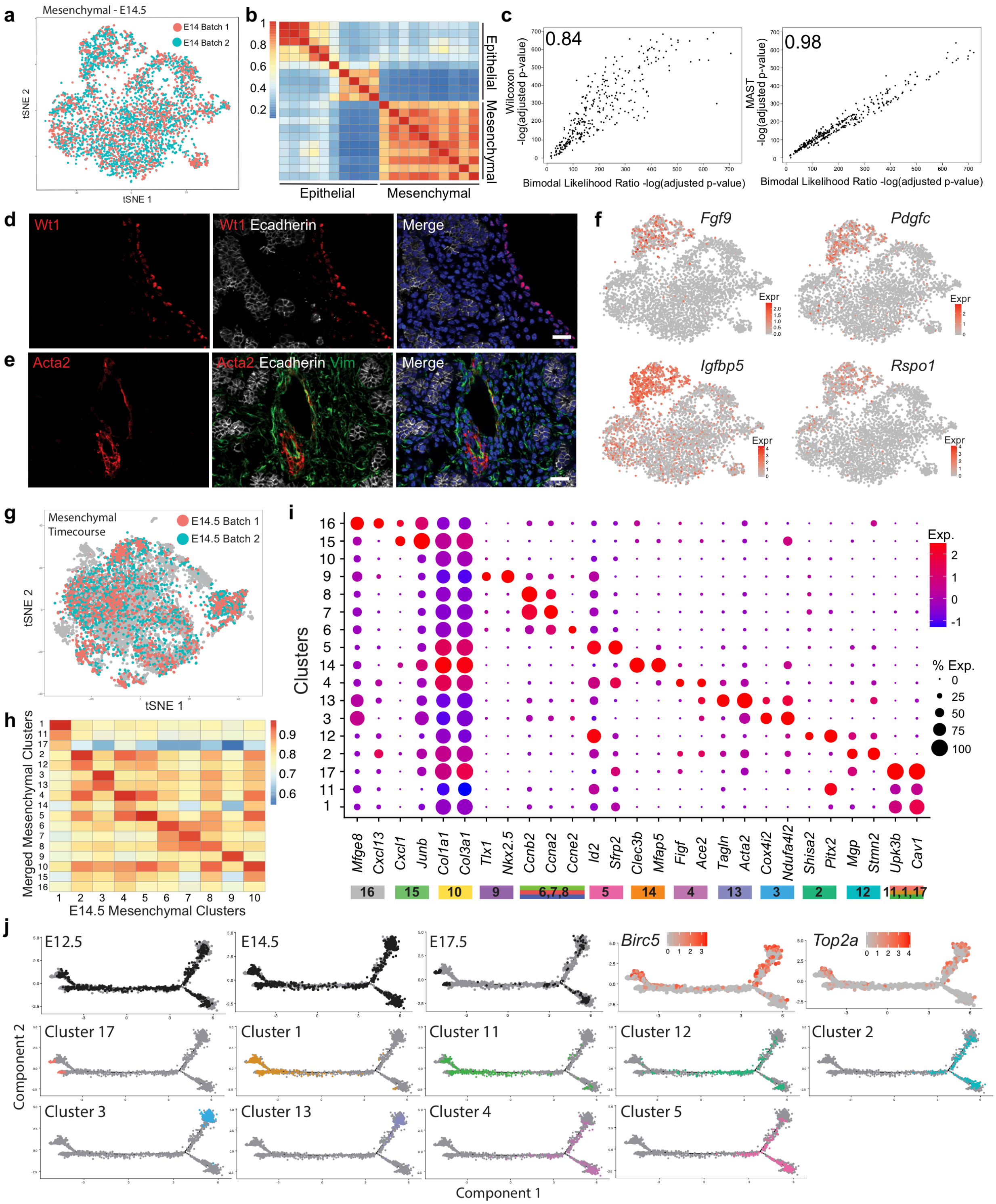

Supplementary Figure 3. Transcriptional signatures and lineage dynamics among mesenchymal populations

(a) t-SNE visualization of E14.5 v1 biological replicates, colored by batch, demonstrating effectiveness of batch correction across mesenchymal cells. (b) Pearson's correlation of E14.5 epithelial and mesenchymal clusters based on average expression of variable genes. (c) Comparison of bimodal likelihood ratio test adjusted p-values to adjusted p-values calculated by either MAST (left panel) or Wilcoxon rank sum (right panel) tests for all greater than 2-fold differentially-expressed genes. Pearson's correlation value is shown in top left corner. (d,e) IF validation of (d) mesothelium (Wt1+) and (e) vascular smooth muscle (Acta2+) cells in E14.5 pancreata. Ecadherin marks epithelium, and Vimentin (Vim) marks mesenchyme. Scale bar: 50 um. (f) Expression of secreted factors by the mesothelium. Color intensity indicates level of expression. (g) t-SNE visualization of merged mesenchymal timecourse dataset. E14.5 biological replicates are colored, serving as a measure of batch correction effectiveness within the merged mesenchymal timecourse dataset. Grey dots represent both E12.5 and E17.5 cells. (h) Correlation of E14.5 mesenchymal populations with merged (E12.5, E14.5 and E17.5) mesenchymal clusters based on average expression of the variable genes from all datasets. Merged populations were matched with E14.5 (Fig. 2) by highest correlation and assigned the same cluster identity (cluster 1-10). Remaining merged clusters were assigned cluster identities 11-17. (i) Dot plot of differentially-expressed genes from each merged mesenchymal cluster. The size of each dot represents the proportion of cells within a given population that expresses the gene; the intensity of color indicates the average level of expression. Colored bars correspond to t-SNE in Fig. 3a (j) Contribution of cells from each timepoint is mapped onto

pseudotime plots. Expression of proliferation markers, *Birc5 and Top2a*, in the pseudotime trajectory. Color indicates level of expression. Contribution of cells from each timepoint is broken down by individual cluster and mapped onto pseudotime plots. Colors correspond to cell clusters in Fig. 3a,e.

Supplementary Figure 4

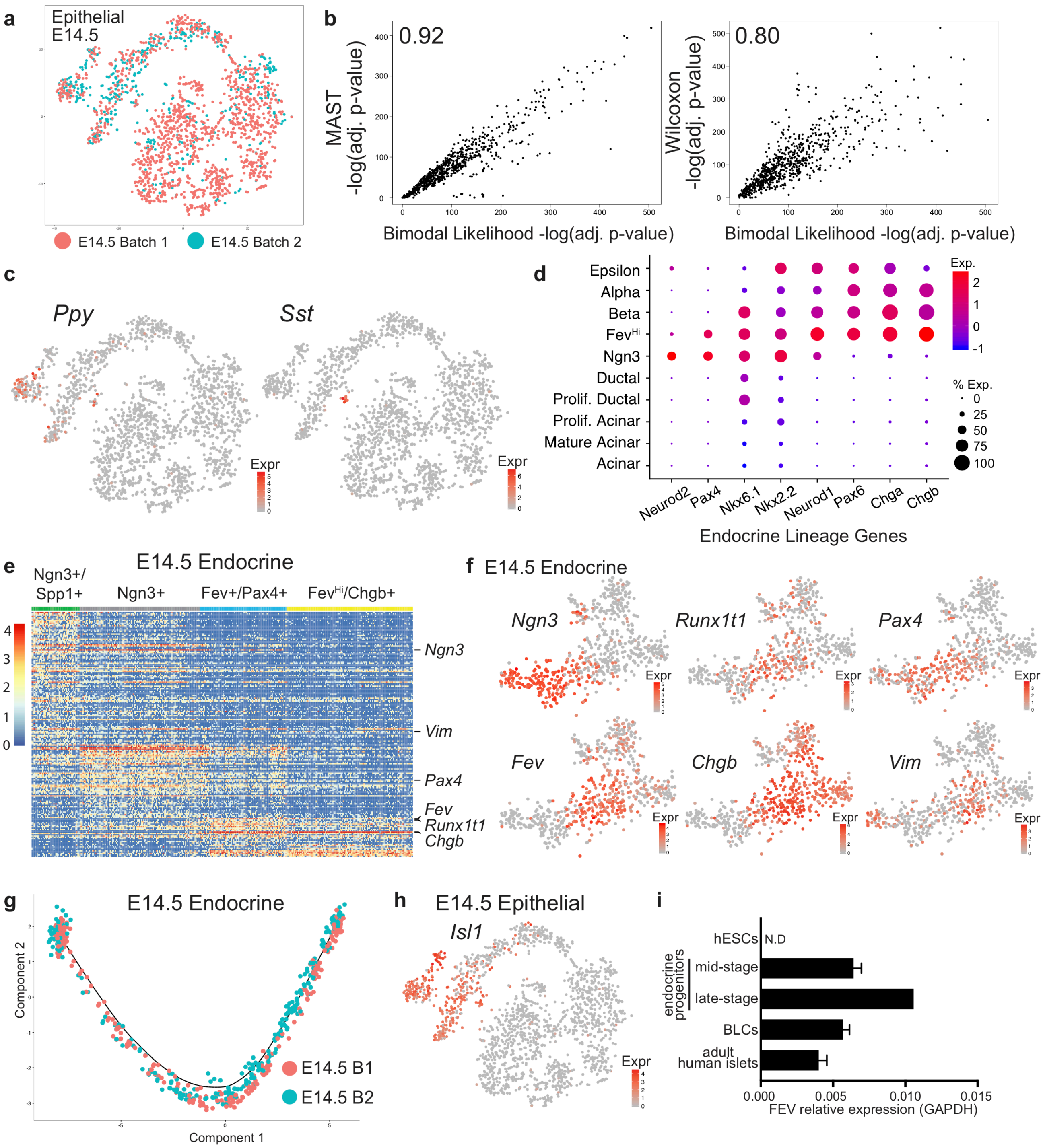

Supplementary Figure 4. Identification of epithelial cell populations in E14.5 pancreas. (a) t-SNE visualization of E14.5 v1 epithelial batches, colored by batch. Significant overlap and most importantly, clusters that include cells from both batches, reflects successful batch correction. (b) Comparison of bimodal likelihood ratio test adjusted p-values to adjusted p-values calculated by either MAST (left panel) or Wilcoxon rank sum (right panel) tests for all greater than 2-fold differentially-expressed genes. Pearson's correlation value is shown in top left corner. (c) Expression maps of *Ppy* and *Sst* hormones within E14.5 epithelial dataset. (d) Dot plot of endocrine lineage genes across the epithelial populations. The size of each dot represents the proportion of cells within a given population that expresses the gene; the intensity of color indicates the average level of expression. (e) Heatmap depicting genes over 2-fold differentially-expressed in Ngn3+ and Fev+ populations. Differentially expressed genes were determined from the endocrine dataset depicted in Fig. 4f and only Ngn3+ and Fev+ populations are shown in the heatmap. (f) Expression of selected markers of early- and late-Fev+ populations in all endocrine cells. (g) Pseudotime ordering of Ngn3+, Fev+/Pax4+, Fev^{Hi}, alpha, and beta cell populations, colored by batch. (h) Expression of *Islet1* (*Isl1*) in E14.5 epithelial cells is largely confined to hormone+ populations. (i) Quantification of *FEV* expression by quantitative RT-PCR in pluripotent hESCs, mid- and late-stage endocrine progenitor cells, beta-like cells (BLCs), and adult human islets. *FEV* expression is normalized to *GAPDH*. Error bars represent standard deviation. N.D = not detected. Bars represent average of three technical replicates from one hESC differentiation.

Supplementary Figure 5

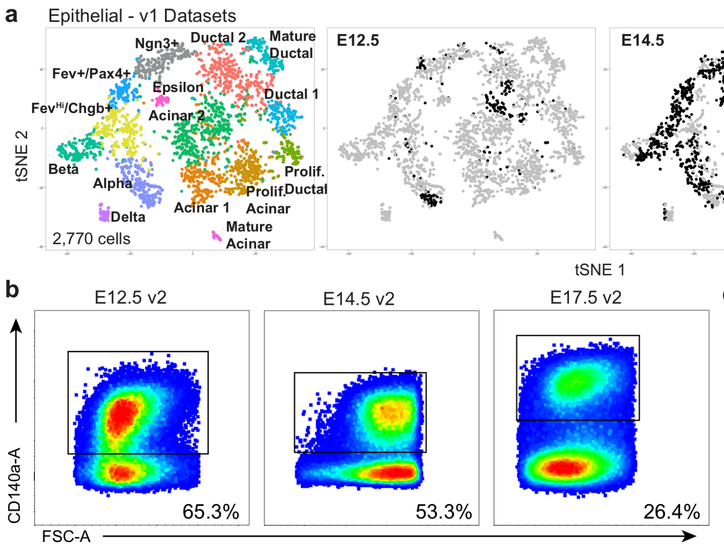

c

	E12.5 v2	E14.5 v2	E17.5 v2 R1 & R2
Cells Captured	13,197	9,196	8,749 & 8,684
Mean reads/cell	25,606	38,976	57,733 & 52,706
Median UMIs/cell	7,944	3,084	10,252 & 9,494
Median genes/cell	2,574	2,466	2,160 & 2,101
Valid Barcodes	97.3%	97.6%	98.3% & 98.2%
Mapped Reads to transcriptome	64.4%	68.6%	71.9% & 72.6%
Sequencing Saturation	33.3%	60.1%	61.7% & 61.8%
Fraction Reads in Cells	89.4%	87.8%	89.9% & 90.4%

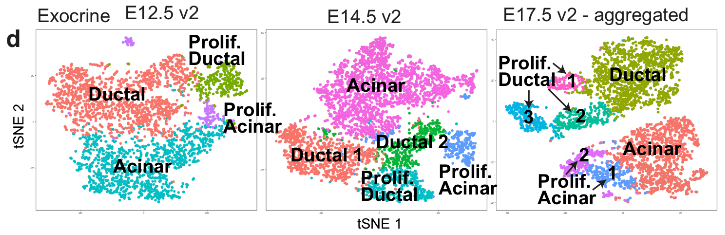

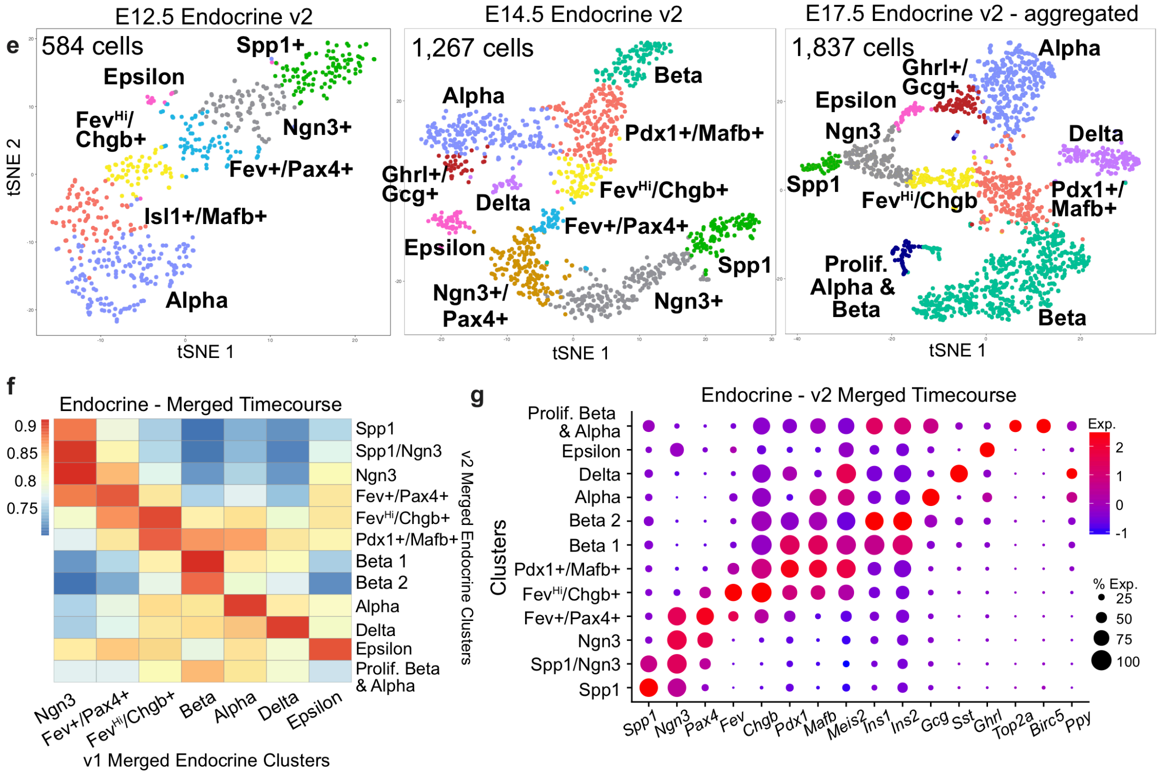

Supplementary Figure 5. Epithelial populations over developmental time. (a) t-SNE visualization of merged version 1 epithelial clusters from E12.5 (n=18 pancreata), E14.5 (n=14 pancreata for batch 1; n=11 for batch 2), and E17.5 (n=8 pancreata). All panels depict the same t-SNE plot. In the far-left panel, cluster identity is denoted by different colors. In the three remaining panels, cells from each indicated timepoint are represented by black dots; all cells from the other timepoints are gray. (b) FACS plots depicting negative selection against CD140a from E12.5 (n=14), E14.5 (n=13), and E17.5 (n=13) pancreata. CD140a-negative cells were used for single-cell sequencing. (c) Quality control statistics for 10X Chromium version 2 single-cell RNA-sequencing runs, referred to as v2 datasets. Two technical replicates of E17.5 cells were run from the same pancreata on two separate wells on the 10X Chromium machine. The two E17.5 runs were aggregated and analyzed as one dataset. (d) Individual t-SNE plots of v2 E12.5, E14.5, and E17.5 (aggregated) exocrine dataset. Clusters are annotated based on gene expression. (e) Individual t-SNE plots of v2 E12.5, E14.5, and E17.5 (aggregated) endocrine dataset. Clusters are annotated based on correlation with v1 datasets and differentially expressed genes. (f) Pearson's correlation among clusters from v1 merged endocrine timecourse and v2 merged endocrine timecourse. (g) Dot plot of top differentially expressed genes for clusters in the v2 merged endocrine dataset. The size of each dot represents the proportion of cells within a given population that expresses the gene; the intensity of color indicates the average level of expression. Clusters correspond to those depicted in t-SNE in Fig. 5h.

Supplementary Figure 6

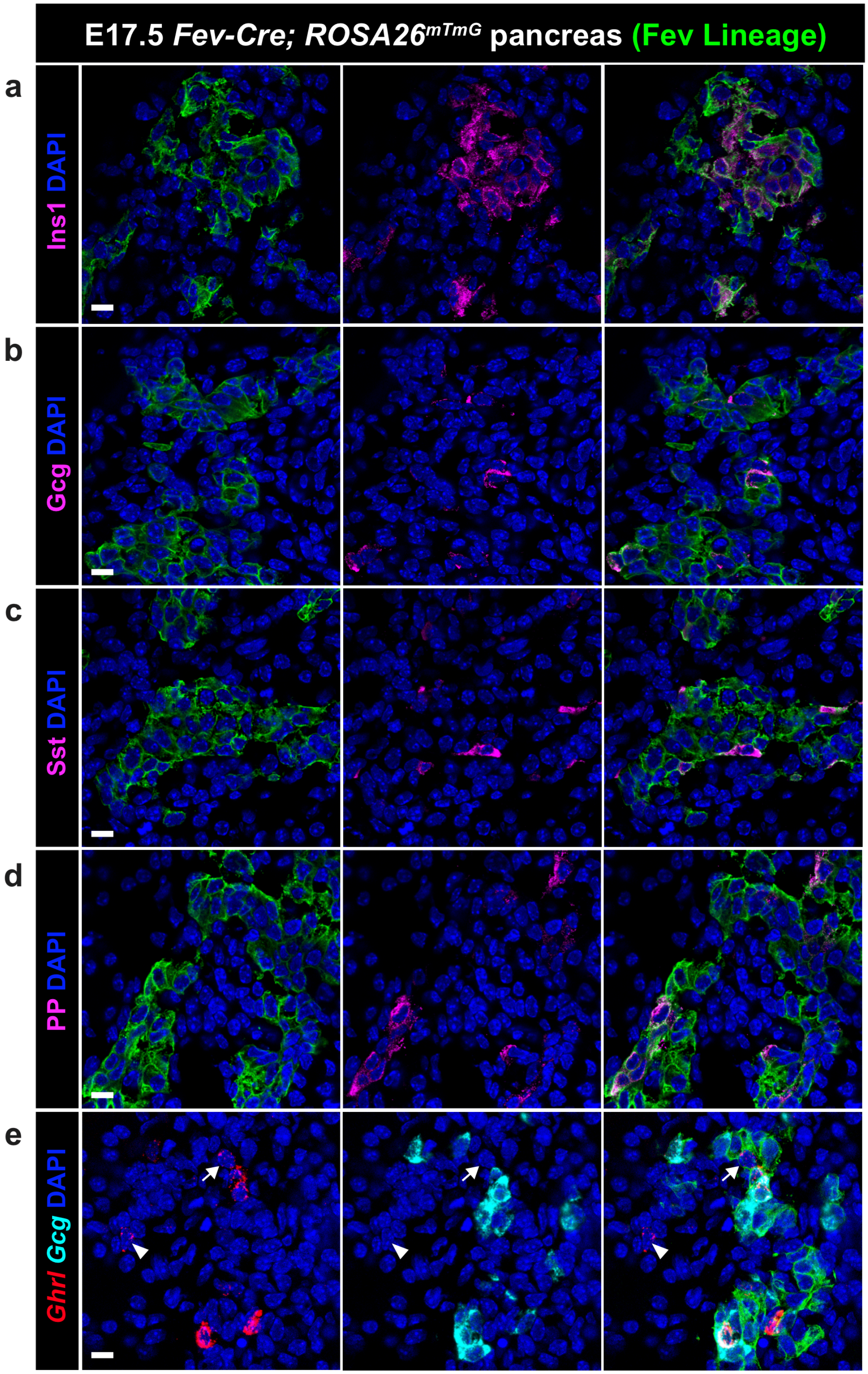

Supplementary Figure 6. Lineage tracing of Fev-expressing cells in E17.5 mouse pancreata *in vivo*. (a-d) Representative images showing immunofluorescence (IF) for hormones Ins1 (100% lineage-labeled), Gcg (100% lineage-labeled), Sst (96.7% lineage-labeled), and Ppy (100% lineage-labeled) in *Fev-Cre; ROSA26^{mTmG}* lineage traced embryos at E17.5 (total n=86 cells from 5 pancreata for Ins1; n=57 cells from 5 pancreata for Gcg; n=30 cells from 5 pancreata for Sst; n=47 cells from 5 pancreata for PP). (e) Multiplexed IF (for membrane-GFP) and fluorescent ISH for *Ghrl* and *Gcg* in *Fev-Cre; ROSA26^{mTmG}* lineage traced embryos at E17.5 (total n=23 cells of 2 pancreata). *Ghrl*+/*Gcg*- cells (47.8% lineage-labeled) represent the epsilon population. Non-lineage labeled epsilon cells are denoted by the arrowheads, and lineage-labeled epsilon cells are denoted by the arrows. Scale bar represents 10um in a-e.

Supplementary Figure 7

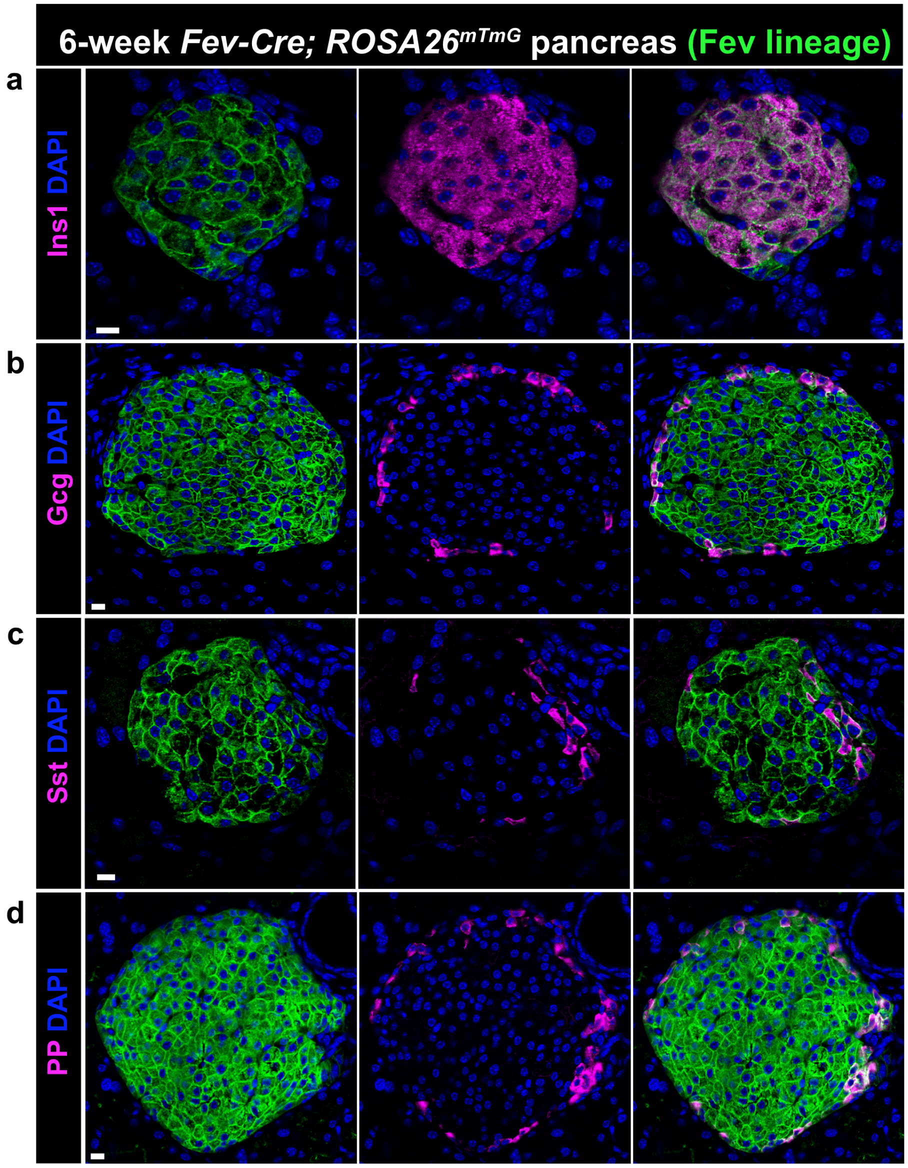

Supplementary Figure 7. Lineage tracing of Fev-expressing cells in adult mouse pancreata *in vivo*. (a-d) Representative IF for adult hormones in 6-week *Fev-Cre; ROSA26*^{mTmG} lineage-traced pancreas. From two animals: n=407 cells for Ins1 (99.6% lineage-labeled); n=120 cells for Gcg (99.0% lineage-labeled); n=116 cells for Sst (97.9% lineage-labeled); n=68 cells for PP (100% lineage-labeled). Scale bar represents 10um in a-d.

Supplementary Figure 8

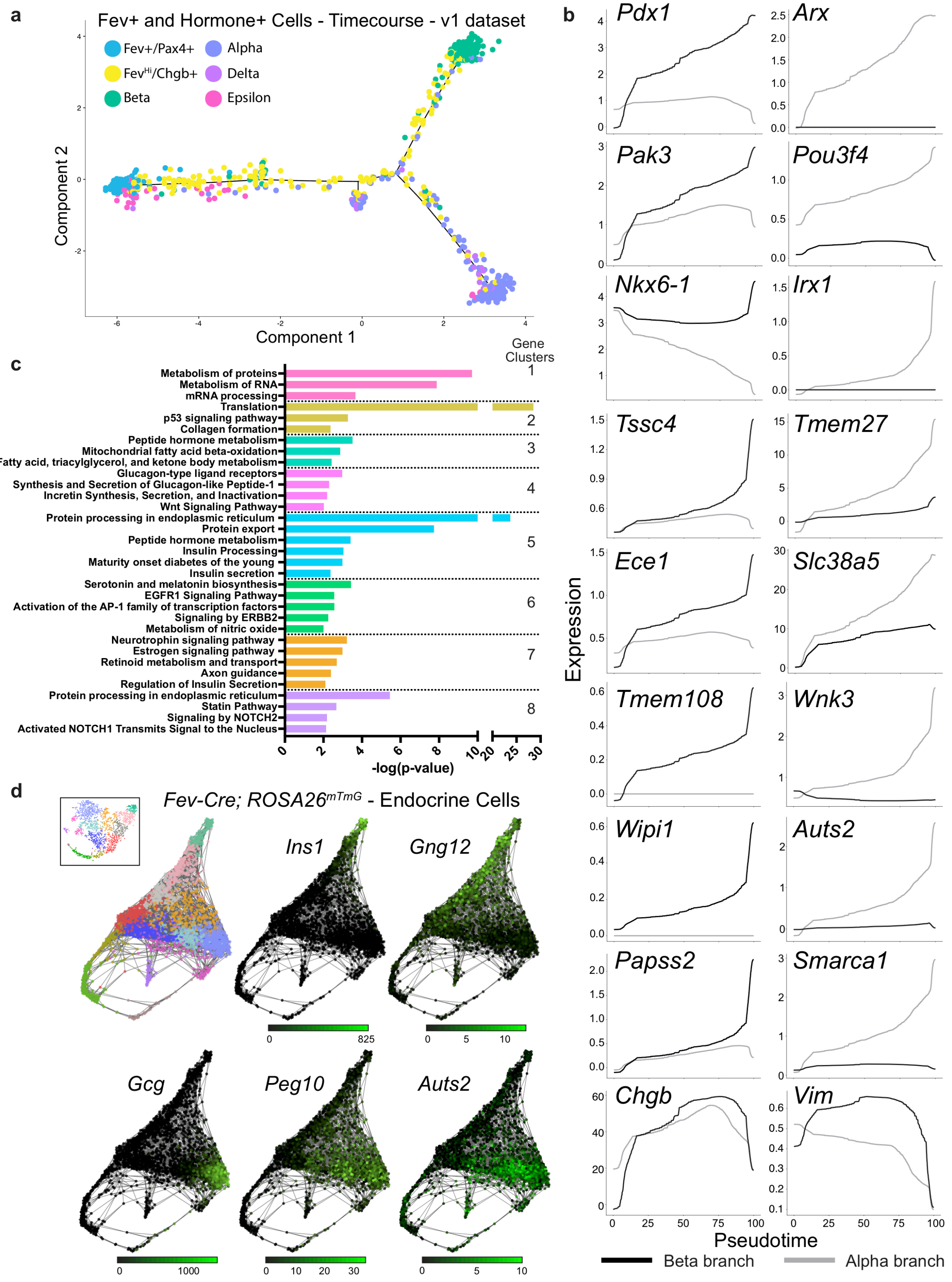

Supplementary Figure 8. Identification of candidate genes and pathways enriched along beta and alpha cell lineages. (a) Pseudotime ordering trajectory of v1 timecourse dataset, including E12.5, E14.5 (batch 1 and batch 2), and E17.5 datasets. (b) Gene expression plots depicting the kinetic curves of individual genes (from Fig. 7b) across pseudotime in the alpha or beta branches. (c) Pathway analysis for clusters of genes from Monocle BEAM analysis. Gene clusters correspond to Figure 7b. (d) SPRING plots for Fev-lineage traced dataset, including all endocrine cells. Colors match those in Fig. 6h and 7a. Expression of selected genes predicted from the BEAM analysis.

Supplementary Figure 9

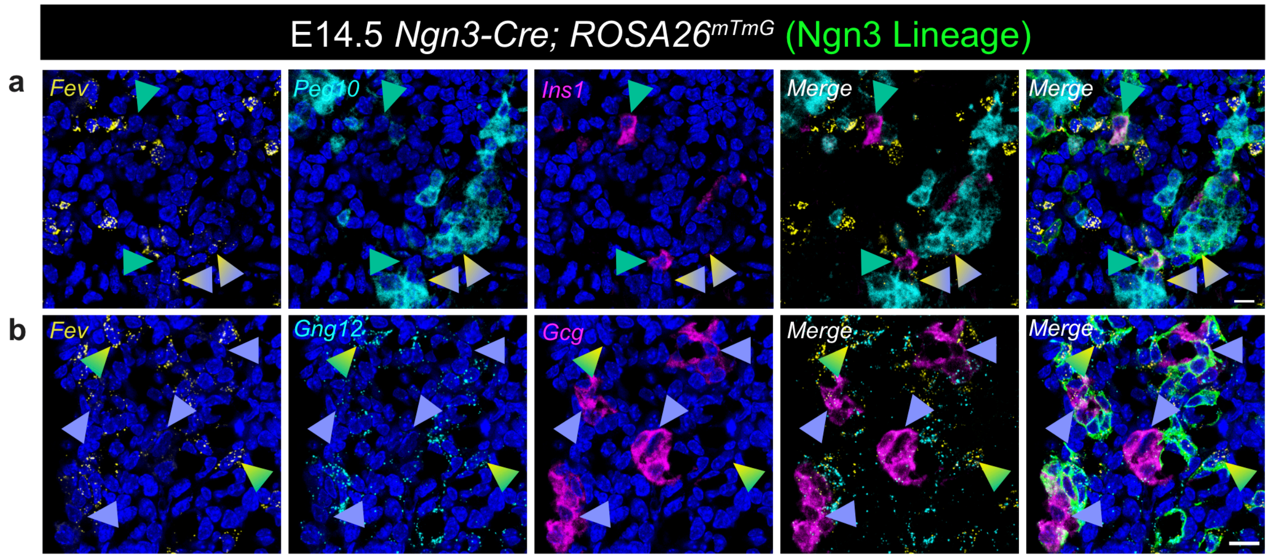

Supplementary Figure 9. Expression of candidate lineage regulators within the endocrine lineage prior to establishment of alpha or beta cell identity. (a) Multiplex fluorescent ISH for *Fev* (yellow), *Peg10* (cyan), and *Ins1* (magenta) in lineage-traced E14.5 *Ngn3-Cre; ROSA26*^{mTmG} pancreas. Indigo gradient arrows highlight lineage-traced *Fev*+/*Peg*10+ cells that do not express *Ins1*. Teal arrows highlight *Ins1*+ beta cells that do not express *Peg10*. (b) Multiplex fluorescent ISH for *Fev* (yellow), *Gng12* (cyan), and *Gcg* (magenta) in lineage-traced E14.5 *Ngn3-Cre; ROSA26*^{mTmG} pancreas. Teal gradient arrows highlight lineage-traced *Fev*+/*Gng12*+ cells that do not express *Gcg*. Indigo arrows highlight *Gcg*+ alpha cells that are not enriched for *Gng12*.
