# Supplementary material for: Lineage dynamics of murine pancreatic development at single-cell resolution
Source: Nat Commun. 2018 Sep 25;9:3922. doi: 10.1038/s41467-018-06176-3 (PMC6156586; doi:10.1038/s41467-018-06176-3)
Supplement: Supplementary file 2 — Description of Additional Supplementary Files [file 41467_2018_6176_MOESM2_ESM.docx]

**Description of Additional Supplementary Files**

File Name: Supplementary Data 1

Description: Differentially-expressed genes of v1 E14.5 batch 1 and batch 2 clusters, including all cells. Genes greater than 2-fold differentially expressed are shown (Gene Name) for each cluster (Cluster ID) in the dataset. The fold change calculated with Seurat’s FindAllMarkers and adjusted p-value from the Bimodal likelihood, Wilcoxon, and MAST tests are indicated for each gene.

File Name: Supplementary Data 2

Description: Differentially-expressed genes of v1 E14.5 batch 1 and batch 2 mesenchymal clusters. Genes greater than 2-fold differentially expressed are shown (Gene Name) for each cluster (Cluster ID) in the dataset. The fold change calculated with Seurat’s FindAllMarkers and adjusted p-value from the Bimodal likelihood, Wilcoxon, and MAST tests are indicated for each gene.

File Name: Supplementary Data 3

Description: Pathway analysis of E14.5 batch 1 and batch 2 mesenchymal clusters. Column “Cluster ID” identifies mesenchymal cluster being analyzed. Calculation of associated p-and q-values were performed using ConsensusPathDB over-representation analysis. Pathways with -log(p-value) greater than 2.0 are shown. Highlighted pathways are those that are displayed in Figure 2d.  Column “source” lists the database from which the indicated pathway derives, and “external_id” lists the pathway identifiers. Columns “members_input_overlap” and “members_input_overlap_geneids” denote the pathway-specific genes and gene IDs that are overrepresented in the specified cluster. The “size” represents the number of genes in each ConsensusPathDB pathway set. The “effective_size” is the corrected size after filtering for genes that are annotated with an ID of the user-specified ID type.

File Name: Supplementary Data 4

Description: Differentially-expressed genes of v1 merged timecourse (E12.5, E14.5 batch 1, E14.5 batch 2, and E17.5) mesenchymal clusters. Genes greater than 2-fold differentially expressed are shown (Gene Name) for each cluster (Cluster ID) in the dataset. The fold change calculated with Seurat’s FindAllMarkers and adjusted p-value from the Bimodal likelihood, Wilcoxon, and MAST tests are indicated for each gene.

File Name: Supplementary Data 5

Description: Differentially-expressed genes of v1 E14.5 batch 1 and batch 2 epithelial clusters. Genes greater than 2-fold differentially expressed are shown (Gene Name) for each cluster (Cluster ID) in the dataset. The fold change calculated with Seurat’s FindAllMarkers and adjusted p-value from the Bimodal likelihood, Wilcoxon, and MAST tests are indicated for each gene.

File Name: Supplementary Data 6

Description: Pathway analysis of E14.5 batch 1 and batch 2 Ngn3+ and Fev^Hi^ clusters. Column “Cluster ID” identifies cluster being analyzed. Calculation of associated p-and q-values were performed using ConsensusPathDB over-representation analysis. Pathways with -log(p-value) greater than 2.0 are shown. Highlighted pathways are those that are displayed in Figure 4e.  Column “source” lists the database from which the indicated pathway derives, and “external_id” lists the pathway identifiers. Columns “members_input_overlap” and “members_input_overlap_geneids” denote the pathway-specific genes and gene IDs that are overrepresented in the specified cluster. The “size” represents the number of genes in each ConsensusPathDB pathway set. The “effective_size” is the corrected size after filtering for genes that are annotated with an ID of the user-specified ID type.

File Name: Supplementary Data 7

Description: Differentially-expressed genes of v1 E14.5 batch 1 and batch 2 endocrine clusters. Genes greater than 2-fold differentially expressed are shown (Gene Name) for each cluster (Cluster ID) in the dataset. The fold change calculated with Seurat’s FindAllMarkers and adjusted p-value from the Bimodal likelihood, Wilcoxon, and MAST tests are indicated for each gene.

File Name: Supplementary Data 8

Description: Differentially-expressed genes of v2 E12.5 exocrine clusters. Genes greater than 2-fold differentially expressed are shown (Gene Name) for each cluster (Cluster ID) in the dataset. The fold change calculated with Seurat’s FindAllMarkers and adjusted p-value from the Bimodal likelihood, Wilcoxon, and MAST tests are indicated for each gene.

File Name: Supplementary Data 9

Description: Differentially-expressed genes of v2 E14.5 exocrine clusters. Genes greater than 2-fold differentially expressed are shown (Gene Name) for each cluster (Cluster ID) in the dataset. The fold change calculated with Seurat’s FindAllMarkers and adjusted p-value from the Bimodal likelihood, Wilcoxon, and MAST tests are indicated for each gene.

File Name: Supplementary Data 10

Description: Differentially-expressed genes of v2 E17.5 exocrine clusters. Genes greater than 2-fold differentially expressed are shown (Gene Name) for each cluster (Cluster ID) in the dataset. The fold change calculated with Seurat’s FindAllMarkers and adjusted p-value from the Bimodal likelihood, Wilcoxon, and MAST tests are indicated for each gene.

File Name: Supplementary Data 11

Description: Differentially-expressed genes of v2 E12.5 endocrine clusters. Genes greater than 2-fold differentially expressed are shown (Gene Name) for each cluster (Cluster ID) in the dataset. The fold change calculated with Seurat’s FindAllMarkers and adjusted p-value from the Bimodal likelihood, Wilcoxon, and MAST tests are indicated for each gene.

File Name: Supplementary Data 12

Description: Differentially-expressed genes of v2 E14.5 endocrine clusters. Genes greater than 2-fold differentially expressed are shown (Gene Name) for each cluster (Cluster ID) in the dataset. The fold change calculated with Seurat’s FindAllMarkers and adjusted p-value from the Bimodal likelihood, Wilcoxon, and MAST tests are indicated for each gene.

File Name: Supplementary Data 13

Description: Differentially-expressed genes of v2 E17.5 endocrine clusters. Genes greater than 2-fold differentially expressed are shown (Gene Name) for each cluster (Cluster ID) in the dataset. The fold change calculated with Seurat’s FindAllMarkers and adjusted p-value from the Bimodal likelihood, Wilcoxon, and MAST tests are indicated for each gene.

File Name: Supplementary Data 14

Description: Differentially-expressed genes of v2 merged timecourse (E12.5, E14.5, and E17.5) endocrine clusters. Genes greater than 2-fold differentially expressed are shown (Gene Name) for each cluster (Cluster ID) in the dataset. The fold change calculated with Seurat’s FindAllMarkers and adjusted p-value from the Bimodal likelihood, Wilcoxon, and MAST tests are indicated for each gene.

File Name: Supplementary Data 15

Description: Gene clusters from BEAM analysis. Column “Cluster ID” identifies gene clusters from BEAM analysis as shown in Figure 7b. The corresponding p-value (“pval” column) and q-value (“qval” column) calculated by BEAM for each gene (“gene_short_name” column) in the given cluster.

File Name: Supplementary Data 16

Description: Pathway Analysis of gene clusters from BEAM analysis. Column “Cluster ID” identifies gene clusters from BEAM analysis used for pathway analysis. Calculation of associated p- (“p-value” column) and q-values (“q-value” column) were performed using ConsensusPathDB over-representation analysis. Pathways with -log(p-value) (“-log(pvalue)” column) greater than 2.0 are shown. Highlighted pathways are those that are displayed in Supplementary Figure 8c.  Column “source” lists the database from which the indicated pathway derives, and “external_id” lists the pathway identifiers. Columns “members_input_overlap” and “members_input_overlap_geneids” denote the pathway-specific genes and gene IDs that are overrepresented in the specified cluster. The “size” represents the number of genes in each ConsensusPathDB pathway set. The “effective_size” is the corrected size after filtering for genes that are annotated with an ID of the user-specified ID type.
